# MiR-277/4989 regulate transcriptional landscape during juvenile to adult transition in the parasitic helminth *Schistosoma mansoni*

**DOI:** 10.1371/journal.pntd.0005559

**Published:** 2017-05-23

**Authors:** Anna V. Protasio, Stijn van Dongen, Julie Collins, Leonor Quintais, Diogo M. Ribeiro, Florian Sessler, Martin Hunt, Gabriel Rinaldi, James J. Collins, Anton J. Enright, Matthew Berriman

**Affiliations:** 1 Wellcome Trust Sanger Institute, Wellcome Genome Campus, Hinxton, United Kingdom; 2 European Bioinformatics Institute, Wellcome Trust Genome Campus, Hinxton, United Kingdom; 3 Department of Pharmacology, UT Southwestern Medical Center, Dallas, TX, United States of America; George Washington University School of Medicine and Health Sciences, UNITED STATES

## Abstract

Schistosomes are parasitic helminths that cause schistosomiasis, a disease affecting circa 200 million people, primarily in underprivileged regions of the world. *Schistosoma mansoni* is the most experimentally tractable schistosome species due to its ease of propagation in the laboratory and the high quality of its genome assembly and annotation. Although there is growing interest in microRNAs (miRNAs) in trematodes, little is known about the role these molecules play in the context of developmental processes. We use the completely unaware “miRNA-blind” bioinformatics tool Sylamer to analyse the 3’-UTRs of transcripts differentially expressed between the juvenile and adult stages. We show that the miR-277/4989 family target sequence is the only one significantly enriched in the transition from juvenile to adult worms. Further, we describe a novel miRNA, sma-miR-4989 showing that its proximal genomic location to sma-miR-277 suggests that they form a miRNA cluster, and we propose hairpin folds for both miRNAs compatible with the miRNA pathway. In addition, we found that expression of sma-miR-277/4989 miRNAs are up-regulated in adults while their predicted targets are characterised by significant down-regulation in paired adult worms but remain largely undisturbed in immature “virgin” females. Finally, we show that sma-miR-4989 is expressed in tegumental cells located proximal to the oesophagus gland and also distributed throughout the male worms’ body. Our results indicate that sma-miR-277/4989 might play a dominant role in post-transcriptional regulation during development of juvenile worms and suggest an important role in the sexual development of female schistosomes.

## Author summary

Schistosomes are parasitic helminths that infect a range of different animals causing great morbidity and some level of mortality among endemic populations in sub-Saharan Africa, southeast Asia and regions of south America. RNA has long been known as a translator of the message between DNA and protein. However, it is nowadays well accepted that RNA roles go beyond that of a translator. Such RNA molecules do not translate DNA into protein and are therefore referred to as non-coding RNAs. Here we study a particular type of RNA molecules called microRNA (miRNA). These small RNA molecules are ~ 19–22 nucleotides (nt) in length and their most characterised function so far is that of regulating the bioavailability of messenger RNA for the production of protein. We call this effect post-transcriptional regulation. Previously, it has been shown that different stages of the *Schistosoma* parasites express different types of miRNAs. Our work focuses in utilising differences in gene expression as the readout of potential post-transcriptional regulation. Using bioinformatics tools we found that members of one miRNA family called miR-277/4989 might be responsible for the change in gene expression observed between juvenile and adult worms. Furthermore, the effect of this miRNA seems to be more prominent in the sexually matured females rather than in the immature “virgin” females.

## Introduction

Schistosomes are parasitic flatworms and causative agents of schistosomiasis (intestinal or urinary depending on the species), and are responsible for more than 200 million cases of human disease across the globe. Three schistosome species infect humans: *Schistosoma haematobium*, *S*. *japonicum* and *S*. *mansoni*. Although *S*. *haematobium* has the greatest clinical impact [1], *S*. *mansoni* is more amenable to maintenance in laboratory conditions [2,3], and therefore is the most studied. In addition, recent advances in functional genomics have been successfully applied to *S*. *mansoni* [4–6] and *S*. *japonicum* [7,8], making functional characterisation of genomic elements more amenable in these parasites.

Unusual among flatworms, schistosomes are dioecious (distinct male and female individuals), and adult worms dwell *in copula* in the blood circulatory system. Females only achieve sexual maturity after pairing with a male worm, lodging in the male’s gynecophoral canal, and laying hundreds of eggs each day. In the absence of a male worm, the female remains sexually immature and stunted in size. The retention of eggs in host tissues drives the pathogenesis of schistosomiasis, mainly characterised by chronic inflammation, fibrosis and formation of granulomas in the liver in the case of intestinal schistosomiais. About half of the eggs traverse the intestinal wall and are excreted with the faeces into the environment. When in contact with fresh water, mature schistosome eggs hatch releasing free-living ciliated larvae (miracidia) that swim seeking snails, the intermediate host, to infect. Within the snail the parasite undergoes two rounds of asexual reproduction, finally releasing the second free-living human-infective larval stage, called cercariae, into the water environment [9]. The cercariae infect the definitive host by penetrating through the skin [10,11] and once inside, transform into obligate parasitic schistosomula via a rapid and irreversible series of morphological changes. The parasites develop and migrate through the circulatory system and after five to six weeks, adult schistosomes reach the portal circulation where males and females pair (reviewed in [12] and [13]). These extreme environmental changes are associated with rapid and specific physiological transformation–such as the parasite surface membranes and carbohydrate coating changing from cercariae to schistosomula [14,15]–accompanied by transcriptional changes [16,17]. Establishment and development of male and female schistosome pairs has been well characterised at both morphological [13] and transcriptional levels [18,19]. However, the underlying molecular cues that trigger such changes are still unknown.

The non-coding RNA component of the *Schistosoma* genomes (*S*. *mansoni* and *S*. *japonicum*) was first described *in silico* [20] followed by reports featuring a combination of experimental and *in silico* approaches [21–29]. Furthermore, the microRNA (miRNA) pathway in *S*. *mansoni* has been predicted using computational methods [30] and more recently, progress in the experimental characterisation of individual components of the pathway [31] as well as the role of individual miRNAs have been addressed [32]. With the increasing availability of high-throughput sequencing technologies (mainly Illumina platforms), the number of publications describing the miRNA, small RNA and non-coding RNA complement of *Schistosoma* spp has risen dramatically, in particular for *S*. *japonicum*. However, the *S*. *japonicum* genome is highly fragmented (25,048 scaffolds in the WBPS9 release available from Wormbase ParaSite—compared to 885 scaffolds for *S*. *mansoni*) making the genome localization of miRNAs and the analysis of their genomic context difficult. What is more, the lack of accurately defined untranslated regions (UTR) makes assessing miRNA-target sites unreliable.

At only ~19–22 nucleotides, miRNAs play a central role in post-transcriptional gene regulation. MiRNAs are encoded in the nuclear genome of most eukaryotic organisms and like protein-coding genes are transcribed by RNA polymerase II, poly-adenylated at their 3’ ends and capped at their 5’ ends. During transcription, a 1kb immature miRNA transcript, called pri-miRNA or primary precursor miRNA, acquires a characteristic stem-loop secondary structure. While still in the nucleus, this structure serves as a target for two proteins, Drosha and DGCR8 (microprocessor complex), which cleave the pri-mRNA at the base of the stem-loop structure producing a pre-miRNA. The pre-miRNA–now 60–80 nt—binds to auxiliary proteins that aid export of the microprocessor complex from the nucleus to the cytoplasm. Once outside the nucleus, Dicer and Argonaut further process the pre-miRNA to form an RNA-induced silencing complex (RISC), which carries the mature miRNA, while the passenger or antisense miRNA (often designated as miRNA*) is degraded. RISC is responsible for directing the miRNA to its target sequence, often (but not exclusively) located in the 3’-UTR of a mRNA, which results in the repression of protein translation or the degradation of the target mRNA molecule (reviewed in [33] and [34]).

MiRNAs have a pivotal role in organism development. For example, in the larval moults of *Caenorhabditis elegans*, the miRNA *lin-4* progressively accumulates in first-stage larvae, down regulating LIN-14 protein and enabling second-stage larvae to develop. Subsequent larval development from L3 to L4 is controlled in part by miRNAs of the *let-7* family [35].

The development, pairing and consequently sexual maturation of schistosomes are of particular interest because they represent the cause of host pathology and transmission of this devastating parasitic disease. To identify whether miRNAs exert an effect on the transcriptome we adopted a non-conventional approach, instead of simply profiling the miRNAs as is commonplace [21,23,24,28,29], we use Sylamer [36], an algorithm that combines transcript expression changes with the presence of potential miRNA recognition sites in well-annotated 3’-UTRs. Our analyses suggest that the sma-miR-277/4989 family of miRNAs dominates the transcriptional landscape changes during the transition from juvenile to adult worm. We show that most of the targets for these miRNAs encode transcription factors, molecules involved in transcriptional activation/repression as well as signalling and proteins associated with adult stem-cell maintenance. Furthermore, a fraction of the targets are differentially expressed between mature, sexually active females and immature “virgin” females suggesting a role in sexual maturation or sexual reproduction.

## Materials and methods

### 1.1. Ethics statement

All animal experiments were conducted under Home Office Project Licence No. 80/2596. All protocols were presented and approved by the Animal Welfare and Ethical Review Body (AWERB) of the Wellcome Trust Sanger Institute. The AWERB is constituted as required by the UK Animals (Scientific Procedures) Act 1986 Amendment Regulations 2012.

### 1.2. Parasite material

Infected *Biomphalaria glabrata* snails were purchased from BioGlab Ltd. Nottingham, UK and cercariae were harvested by exposure of infected snails to light for two hours in aquarium-conditioned water. *S*. *mansoni* intra-mammalian stages were collected from Balb/c mice previously infected with 300-pooled cercariae. For single sex infections (to retrieve unpaired male and female worms), mono-miracidia snail infections were performed and each snail tested for the production of male or female cercariae, by sex-specific PCR [37]. Balb/c mice were infected separately with either male or female cercariae. Juvenile and adult worms were recovered via perfusion of mouse circulatory system [2] at the indicated times post-infection (see Results). Male and female worms were separated, washed in DMEM, concentrated and stored in Trizol reagent (Invitrogen, UK) at -80°C.

### 1.3. RNA extraction, RNAseq library preparation and sequencing

RNA was isolated from samples stored in Trizol reagent (Invitrogen, UK) following manufacturer instructions. RNAseq libraries were prepared from 1ug of total RNA using TruSeq RNA Library Preparation Kit (Illumina, UK). All samples were processed with three biological replicates–worms from one mouse representing one biological replicate. Libraries were multiplexed and sequenced on Illumina HiSeq 2500 with 100 bp paired-end reads. Sequencing data was submitted to ArrayExpress under accession number E-ERAD-478.

### 1.4. Processing of poly-A enriched RNAseq sequencing reads and differential expression analysis of paired / unpaired male and female worms

RNAseq data from juvenile and adult, male and female schistosome worms were mapped to the reference *S*. *mansoni* assembly version 5.2 of the genome assembly [38] using Tophat2 (default parameters except: -g 1—library-type fr-firststrand -a 6 -i 10 -I 40000—microexon-search—min-segment-intron 10—max-segment-intron 40000). The resulting binary alignment mapping (BAM) files were sorted using Samtools [39] and reads per transcript calculated using HTseq-count [40] (parameters: -f bam -a 30 -t CDS -s reverse -m union). The GTF file used to calculate reads per transcript can be found in Supplementary S1 Dataset; only features starting with “Smp” (corresponding to protein coding genes) were taken into consideration. After mapping and counting, differential expression analysis was performed using DESeq2 [41] in the R environment [42]. Pairwise differential expression values for male and females worms (from mixed infections) between juveniles (21 days post infection d.p.i.) and adult worms (38 d.p.i.) were calculated and used as input for Sylamer (see below). Time-course expression data for male and female, paired and unpaired worms was generated using likelihood ratio test incorporated in DESeq2 package.

### 1.5. UTR prediction and experimental validation

RNAseq data generated from polyadenylated-enriched samples was used to improve the annotation of 3’-UTRs in the *S*. *mansoni* genome. BAM files generated with Tophat2 were merged using Samtools [39] and used as input for Cufflinks [43,44] (using default parameters except: -p 16—library-type fr-firststrand). To generate UTR sequences, RNAseq data and existing annotation were combined using a script developed in-house (https://github.com/sanger-pathogens/Assembly_tools/tree/master/assembly_tools/annotate_utrs_using_cufflinks). Briefly, for each existing gene, the script takes an intersecting annotation from the cufflinks GTK output file and extends the existing gene model using the provided annotation, labelling the new part of the annotation as a UTR. If the potential UTR happens to overlap a second gene, then it is not used. As a result, 3,321 3’-UTRs from a total of 7,373 3’-UTRs were updated using this method. Likewise, 4,081 5’-UTRs from a total of 7,271 5’-UTRs were updated. The total number of protein-coding genes is 10,841. Note that this version of the annotation is a “snapshot” and may not coincide with the current version in http://www.genedb.org/Homepage/Smansoni or http://parasite.wormbase.org/Schistosoma_mansoni_prjea36577/Info/Index/.

A selection of twelve 3’-UTRs with lengths ≥ 600 bp, and a range of RPKM expression values between 9–3,600 in adult worms, were chosen for validation by PCR (Supplementary S1 Fig). Primers were designed using primer3 batch service [45] using default parameters except amplicon length, which was set to 500 bp. PCR reactions were carried out using Qiagen Fast Cycling PCR Mix (Qiagen, UK) using standard conditions with annealing temperature 55C and mix sex adult cDNA as template. The list of primers is shown in Supplementary S1 Table.

### 1.6. Sylamer analysis

Sylamer [36] is a tool to identify miRNA regulation effects from a list of differentially expressed genes, independently from miRNA measurements. It can either be used to suggest candidate miRNAs for follow-up analysis in the absence of miRNA measurements, or to confirm that putative miRNA target genes are shifted concordantly with miRNA expression changes when miRNA measurements are present. The latter is the case here. It has previously been used, among many more applications, to identify the role of miRNAs in germcell tumors [46], the effect of a miRNA seed mutation on gene expression [47], and identification of miRNA targets in murine Dgcr8-deficient embryonic stem cell lines [48]. Sylamer’s *modus operandi* is briefly described here, for full details refer to [36]. Several nucleotide words in the eight-nucleotide stretch (8-mer) at the 5' end of a miRNA are core determinants of miRNA binding [34,49]. These are the 8-mer itself, the two 7-mers, the core 6-mer, and the leading 6-mer. The nucleotide sequences that are complementary to these miRNA seed words are called Seed Complementary Regions (SCR). Sylamer considers a list of differentially expressed genes and tests the hypothesis that SCRs in 3’-UTRs are shifted towards one end of the gene list against the null hypothesis that these sites are homogeneously distributed throughout the gene list. It does so whilst taking into account UTR length and correcting for composition biases, using a hypergeometric model of nucleotide word occurrences in UTRs. Analogously to Gene Set Enrichment Analysis [50], Sylamer employs a moving rank cut-off and can hence detect shifts of SCRs that are dispersed to a greater or lesser extent.

#### Sylamer plots construction

**Fig 1:**
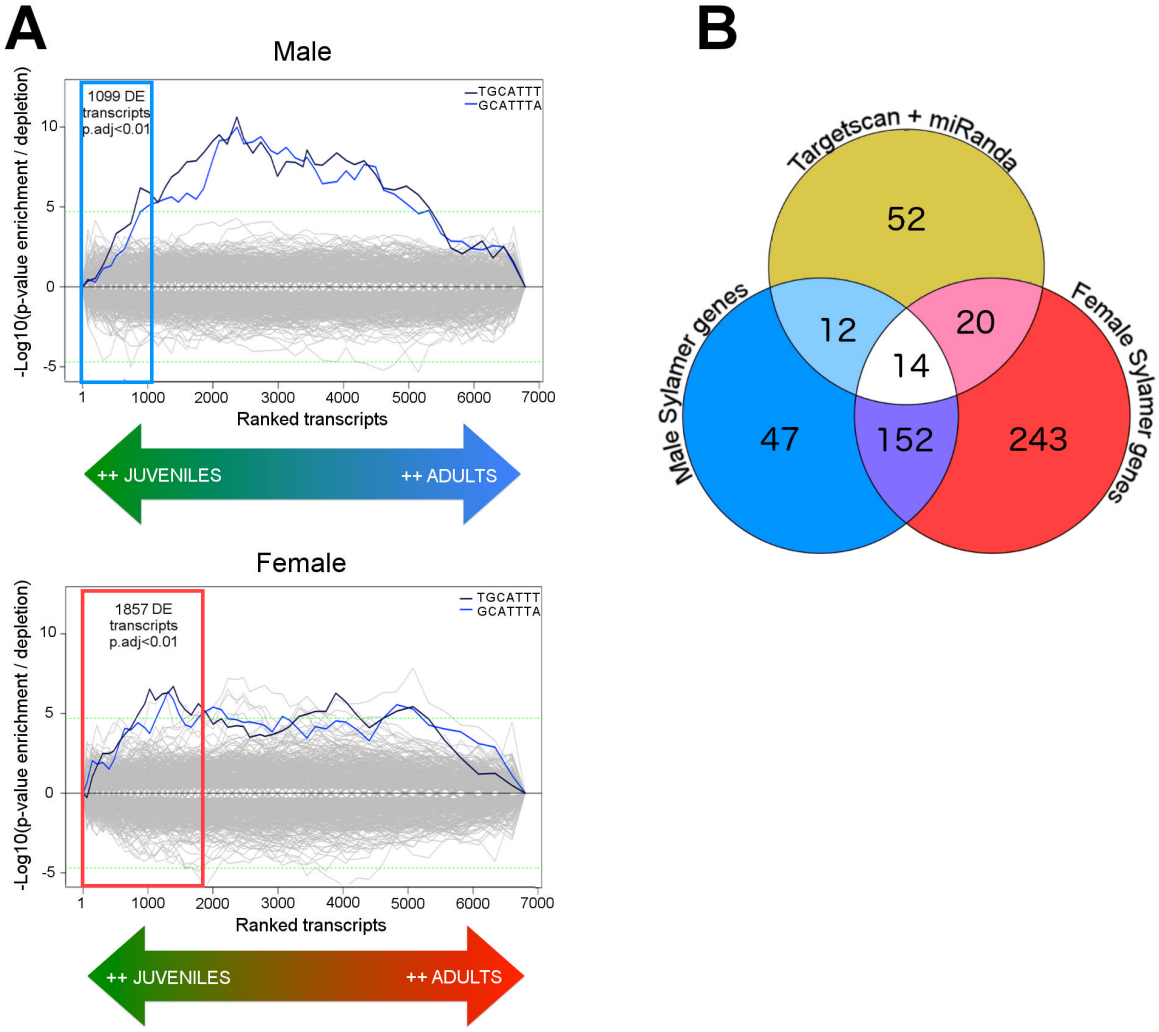
MiRNA target prediction based on both miRNA-unaware and miRNA-guided approaches. (A) Sylamer enrichment landscape plots for 7mers in male (top) and female (bottom) expression data. The x-axis represents a list of transcripts, ranked from more expressed in juveniles to more expressed in adults. The y-axis represents the significance values acquired for each 7mer at each position in the ranked list of transcripts. Coloured boxes represent the fraction of transcripts significantly (adjusted p-value < 0.01) differentially expressed between juvenile and adult worm as found using DESeq2. These transcripts were subsequently filtered based on the presence of the 7mers TGCATTT or GCATTTA as found by Sylamer. The resulting sets are referred to as Male and Female Sylamer genes. (B) Venn Diagram showing the intersection of Male and Female Sylamer genes with schistosome-conserved miRNA targets as found using TargetScan with conservation + miRanda. The overlap represents transcripts with highly conserved sma-miR-277 target sites across the three *Schistosome* spp (S. *mansoni*, *S*. *haematobium* and S. *japonicum*) that are also significantly down regulated during worm development.

All information produced by a Sylamer run can be summarised in a single plot, as a collection of lines where each line represents a single SCR. In a single Sylamer run, the word length is kept constant, so different plots are obtained for SCRs of length 6, 7 and 8, respectively. A single line in a Sylamer plot describes a single SCR as follows: at different cut-offs in the list of UTRs, a hypergeometric test is performed considering the summed total length of the UTRs up to that cut-off (representing the number of all SCRs), the summed total length of all UTRs, the number of occurrences of the particular SCR up to the cut-off, and the total number of occurrences of the particular SCR in all UTRs. If the SCR is enriched in the subset of UTRs, the -log10(p-value) is drawn (hence at the positive y-axis). If is instead depleted, the log10(p-value) is drawn (at the negative y-axis). Multiple cut-offs are tested and a large number of SCRs are tested. On the one hand, this requires multiple testing correction, on the other hand this allows a single SCR result to be evaluated against the background of plotted lines for all SCRs. A Bonferroni-adjusted significance threshold of 0.05 (as drawn in Fig 1A and 1B) typically delineates and encloses the background distribution with significant SCRs jutting out.

Additional significant SCRs above the Bonferroni-adjust threshold may occur in a Sylamer plot. This can be due to several causes. Firstly, multiple regulatory elements besides SCR are present in UTRs, such as poly-A and ARE (AU-rich element) signals. MiRNA SCRs may share such motifs. Secondly, repeated sequence fragments in UTRs of related genes may cause sharp spikes in Sylamer plots, and stretches of low complexity sequence can cause significant results for SCRs matching such a sequence. This is overcome (as done here) by pre-processing the UTRs to remove low complexity stretches with DUST [51] and repeated sequence fragments with the RSAT [52] interface to the Vmatch program (http://www.vmatch.de). Finally, a significant SCR signal can cause words that are very close to it to piggyback the signal and achieve elevated significance. This is then evident from the words involved.

#### Sylamer result intergration over different word lengths

Sylamer was run with different word lengths because miRNA determinants of binding can be found among words of different length [34,36]. It is possible to run Sylamer with different word lengths, as we have done here. This provides greater insight but also requires interpretation of multiple resources. We additionally apply the procedure described in [46] to integrate Sylamer results for different word lengths were integrated into a single score for a given miRNA seed region, using a previously described procedure [46]. By integrating results, sensitivity was increased. The procedure assigned miRNAs to groups, each group defined by a common SCR of length eight. A single score was assigned to each group by considering the Sylamer result (line) for each of the constituent 6-mer, 7-mer and the 8-mer itself, obtained from different Sylamer runs. These lines (log10 transformed p-values) represent a normalised view (via the hypergeometric test) of word enrichment. The constituent lines were added together, after which the maximal amplitude of the summed result was taken as a single score. As demonstrated in [46] the resulting scores narrowly follow an extreme value distribution. The extreme value distribution parameters were estimated with the R /evd/ package [53] using the 95% quantile of the data and then obtain empirical p-values using these parameters. In the absence of a null hypothesis these empirical p-values can be interpreted/translated as the percentile range into which a score falls under the modeled distribution, i.e. a p-value of 0.0001 would correspond to the top 0.01 percentile.

### 1.7. miRNA target prediction (miRanda and TargetScan) and gene ontology enrichment

Targets were predicted in the 3’-UTRs of 4,851 genes for previously reported *S*. *mansoni* and *S*. *japonicum* miRNAs and the novel miRNA from the present study. First, miRanda (v 3.3a, ref. [54]) was run with default parameters against the predicted set of *S*. *mansoni* 3’-UTRs. Second, TargetScan (v 6.0, ref. [55]) was used to search the same 3’-UTRs dataset; the script targetscan_60.pl was run with default parameters, using the seed sequences of each *Schistosoma* spp. miRNA (from miRBase, release 21, ref. [56]) and alignments of orthologous 3’-UTRs (see below) between *S*. *mansoni*, *S*. *japonicum* and *S*. *haematobium*. Using custom scripts, only *S*. *mansoni* TargetScan target site predictions conserved among the three Schistosoma species were retained, i.e. requiring the target prediction to be present on the same exact aligned location on the all orthologous 3’-UTRs. Further to this, only a high-confidence set of predictions found by both TargetScan and miRanda were retained. Except for *S*. *mansoni*, current 3’-UTR predictions for the other two Schistosoma species used were not reliable; therefore new 3’-UTR predictions for *S*. *haematobium* and *S*. *japonicum* were created based on orthology with *S*. *mansoni*. For each *S*. *mansoni* gene the mRNA isoform was selected with the longest spliced sequence, and for genes with annotated 3’-UTRs, orthologous genes in *S*. *japonicum* and *S*. *haematobium* were identified using a custom-made EnsemblCompara database [57]. For each *S*. *japonicum* or *S*. *haematobium* orthologue, a predicted 3’-UTR was created with the same length of the 3’-UTR of the respective *S*. *mansoni* orthologous gene (up to maximum of 5 kb or to the end of underlying sequence scaffold). These predicted sets of 3’-UTRs were termed “orthologous 3’-UTRs”, even though orthology is based on the underlying gene product to which they correspond. Alignments of orthologous 3’-UTR sets for the TargetScan predictions were produced with MUSCLE v3.8.31 [58], with default parameters. Assemblies and gene sets of the three species used for accessing conservation of miRNA target predictions can be downloaded at WormBase ParaSite release 4 (http://parasite.wormbase.org/ftp.html): *S*. *haematobium* ‘S.haematobium.v3.0’ from the University of Melbourne, and *S*. *japonicum* ‘ASM15177v1’ from the Chinese National Human Genome Center. The *S*. *mansoni* assembly used here is the most updated version reported previously [38].

Gene Ontology enrichment analysis was performed using the TopGO package [59].

### 1.8. Processing of small RNA-seq libraries and genomic localisation of sma-miR-277/4989 cluster

We used previously generated small RNA-seq libraries from schistosomula stages of *S mansoni* to assist our description of the sma-miR-277 locus (European Nucleotide Archive study PRJEB3190). Kraken [60] was used to remove adapter contamination from libraries and collapse identical reads into single sequences while maintaining annotated depth information (Supplementary S2 Table). Reads were then mapped against each other using BLASTN [61,62] to determine all pairwise similarities between reads allowing up to two mismatches (E-value < = 0.1). A pairwise similarity matrix was used to cluster reads using MCL [60,63]. Multiple sequence alignment of each cluster was performed using Clustal Omega [64]. From each cluster, a ‘sentinel’ read was chosen with the highest depth and mapped to the reference genome using Bowtie [65], allowing up to two mismatches.

After identifying the genomic location for the candidate and in order to obtain the putative miRNA precursor sequence, the sequences were extended in both directions. The first extension took 50nt upstream and 100nt downstream while the second extension took 100nt upstream and 50nt downstream of the cluster. Secondary structures of these putative miRNA precursors were then assessed using RNAfold from the Vienna package [66], and structures discarded with minimum free energy (MFE) > -20 kcal/mol. For each cluster, the extended sequence with the lowest associated MFE structure was retained.

### 1.9. RT-qPCR / miRNA expression analysis

Real-time quantitative PCRs were performed across two life cycle stages of *S*. *mansoni* representing juvenile (28 days post-infection, d.p.i.) and adult worms (49 d.p.i.) using TaqMan Small RNA Assays (Supplementary S1 Text) purchased from Applied Biosystems (Life Technologies, UK). Reverse transcription and quantitative PCR experiments were performed according to manufacturer’s instructions in a StepOnePlus RT-qPCR machine (Applied Biosystems, Life Technologies, UK). *S*. *mansoni* U6 was used as the endogenous control [20] (gene entry sma.U6.1.1 in http://www.genedb.org/Homepage/Smansoni) and fold changes to miRNA expression were estimated using the delta-delta-Ct method [67].

### 1.10. Whole mount and fluorescence *in situ* hybridisation of miRNAs

Forty-two to 49 d.p.i. male and female worms from mixed infections were retrieved from infected mice, as described above, and processed using previously published protocols for *in situ* hybridization [68]. Parasites were hybridized with 21–22 nt antisense LNA-DNA probes conjugated to Digoxigenin (Exiqon, Denmark) at a final concentration between 6.5–50 nM. *Arabidopsis thaliana* ath-miR-159a or scrambled sequences were used as negative control. For FISH to detect *prohormone convertase 2* (Smp_077980) or Tegumental cells, we synthesized FITC-conjugated probes generated from *in vitro* transcription of cloned cDNA. To robustly detect tegumental cells we employed a mixture of four FITC-conjugated antisense probes targeting the following tegument-specific mRNAs: *calpain* (Smp_214190), *gtp -4* (Smp_105410), *annexin* (Smp_077720), and *npp-5* (Smp_153390) (ref: [69–73]).

## Results

### 2.1. Target-prediction suggests sma-miR-277/4989 are prominent post-regulatory miRNAs in developing juvenile worms

In our analysis, Sylamer [36] was used to search for enriched short sequences, corresponding to potential miRNA target sites, from a list of genes differentially expressed between juvenile and adult male and female worms. Sylamer finds enriched “words” (in our case, Seed Complementary Regions or SCR) in the 3’-UTRs of transcripts with similar expression profiles (i.e. up-regulated or down-regulated). The advantage of this method is that potential targets can be identified without prior knowledge of the specific miRNAs that affect the transcriptome. Sylamer indicated that the 7mers TGCATTT/GCATTTA, corresponding to the SCR of miR-277 family, were significantly enriched in male (p-value = 1.62E-13, Supplementary S3A Table) and female (p-value = 1.22E-06, Supplementary S3B Table) genes that were more highly expressed in juvenile worms compared to adults (Fig 1A).

Genes with 3’-UTRs containing the 7mers TGCATTT/GCATTTA were selected from the list of transcripts significantly upregulated (adjusted p-value ≤ 0.01) in juveniles compared to adult worms, using genes found in males (n = 1,099) and females (n = 1,857) separately. Among these transcripts, 225 male and 429 female transcripts contained the 7mers TGCATTT/GCATTTA in their 3’-UTRs (as predicted by Sylamer) and therefore were regarded as potential targets for miRNAs of the sma-miR-277 family. We refer to these groups as male and female “Sylamer genes” (Supplementary S2 Fig).

Secondly, we employed miRanda [54] and TargetScan with conservation evidence [55] to perform miRNA-guided predictions of potential targets in the transcriptome. This latter approach is based on curated three-way alignments of 3’-UTR sequences from orthologous genes from *S*. *mansoni*, *S*. *haematobium* and *S*. *japonicum* (see Materials and Methods). Unlike Sylamer, the combined results of miRanda (Supplementary S4E Table) and TargetScan with conservation (Supplementary S4F Table) only take into account known miRNA seeds in the 3’-UTRs and not the expression of their transcripts.

We further refined this list by selecting those Sylamer genes that also have conserved target sites across the three schistosome species (Fig 1B) as identified by combining miRanda [54] and TargetScan with conservation [55]. Notably, miRanda and TargetScan with conservation alone found 98 potential targets for the sma-miR-277 family in the Schistosoma transcriptome (Supplementary S4 Table), considerably higher than for any other miRNA in this study. Of these, 46 genes were also “Sylamer genes” (26 male and 34 female gene with 14 shared between the two genders—Fig 1B and Table 1), we called these “high confidence targets”. By performing a hypergeometric test, we calculated the probability that these overlaps are significant, using as background the 1,476 protein-coding genes that possess at least the core sma-miR-277 6mer GCATTT. Our results show that there is significant enrichment in the overlaps between TargetScan+Miranda vs. Male Sylamer genes (p-value < 0.002) and also between TargetScan+Miranda vs. Male (p-value < 1e-50) while the overlap between TargetScan+Miranda vs. Female Sylamer genes was not significant.

**Table 1:** High confidence targets of the sma-miR-277 family identified with a combined approach (Sylamer, miRanda and TargetScan with conservation). F = female; M = male.

Gene	Product description	gender
Smp_163500	Protein Sprint	F
Smp_052420	centaurin gamma 1A	F
Smp_072140	Rho2 GTPase	F
Smp_042300	PUR alpha protein	F
Smp_009630	homeobox protein SIX6	F
Smp_172240	stress activated protein kinase JNK	F
Smp_048560	Four and a half LIM domains protein 2	F
Smp_031270	hypothetical protein	F
Smp_041760	chromodomain helicase DNA binding protein 2	F
Smp_127440	Serine rich adhesin for platelets	F
Smp_173900	pyruvate dehydrogenase phosphatase	F
Smp_015530	hypothetical protein	F
Smp_156480	hypothetical protein	F
Smp_078270	mps one binder kinase activator 2 like	F
Smp_199010	ras GTP exchange factor son of sevenless	F
Smp_210730	prolyl oligopeptidase (S09 family)	F
Smp_141980	cAMP specific 3',5' cyclic phosphodiesterase 4D	F
Smp_194640	serine:threonine protein kinase MARK2	F
Smp_198380	Argonaute	F
Smp_143070	kelch 2, Mayven	F
Smp_105970	Caprin 1	M
Smp_043100	Heterogeneous nuclear ribonucleoprotein A1	M
Smp_155060	phosphatase 2a inhibitor i2pp2a	M
Smp_072110	programmed cell death involved protein	M
Smp_211100	inner nuclear membrane protein man1	M
Smp_123630	ubiquitin carboxyl terminal hydrolase 49	M
Smp_100090	high mobility group protein B1	M
Smp_063040	actin 6a	M
Smp_169320	protein phosphatase 1g	M
Smp_006320	KH domain containing, RNA binding, signal	M
Smp_044810	zinc finger CCCH domain containing protein 14	M
Smp_008830	SWI:SNF related, matrix associated, actin	M
Smp_167320	serine threonine rich antigen	M/F
Smp_129840	hypothetical protein	M/F
Smp_173380	tyrosine kinase	M/F
Smp_147950	bromodomain containing 2	M/F
Smp_139530	tumor protein p73	M/F
Smp_172700	cyclin dependent kinase 6	M/F
Smp_158610	TATA binding protein associated factor 172	M/F
Smp_079650	chromodomain helicase DNA binding protein 4	M/F
Smp_021340	max dimerization protein 3	M/F
Smp_163240	t box transcription factor tbx2	M/F
Smp_200020	FAD NAD binding oxidoreductase	M/F
Smp_149000	protein phosphatase 2C	M/F
Smp_166070	ras specific guanine nucleotide releasing factor	M/F
Smp_165280	protein groucho	M/F

Gene Ontology (Biological Processes) enrichment analysis of the male and female high confidence targets shows significant enrichment (p-value < 0.01) for “chromatin assembly or disassembly” (four genes: Smp_155060, Smp_041760, Smp_158610, Smp_079650), “regulation of transcription, DNA-templated” (11 genes: Smp_042300, Smp_009630, Smp_147950, Smp_139530, Smp_041760, Smp_158610, Smp_079650, Smp_021340, Smp_163240, Smp_100090, Smp_008830) and “protein dephosphorylation” (three genes: Smp_173900, Smp_169320, Smp_149000) categories (Supplementary S5 Table).

### 2.2. Genomic localisation, structural characterisation of sma-miR-277 and identification of sma-miR-4989

Using *Drosophila melanogaster* dme-miR-277 as a query in the miRBase database (Release 21, [56]) we found full-length sequence similarity and seed sequence (nucleotides 2–7) identity with sja-miR-277 from *S*. *japonicum* [28] as well as with two *Echinococcus* spp miRNA sequences namely egr- or emu–miR-277 and miR-4989 [74].

**Fig 2:**
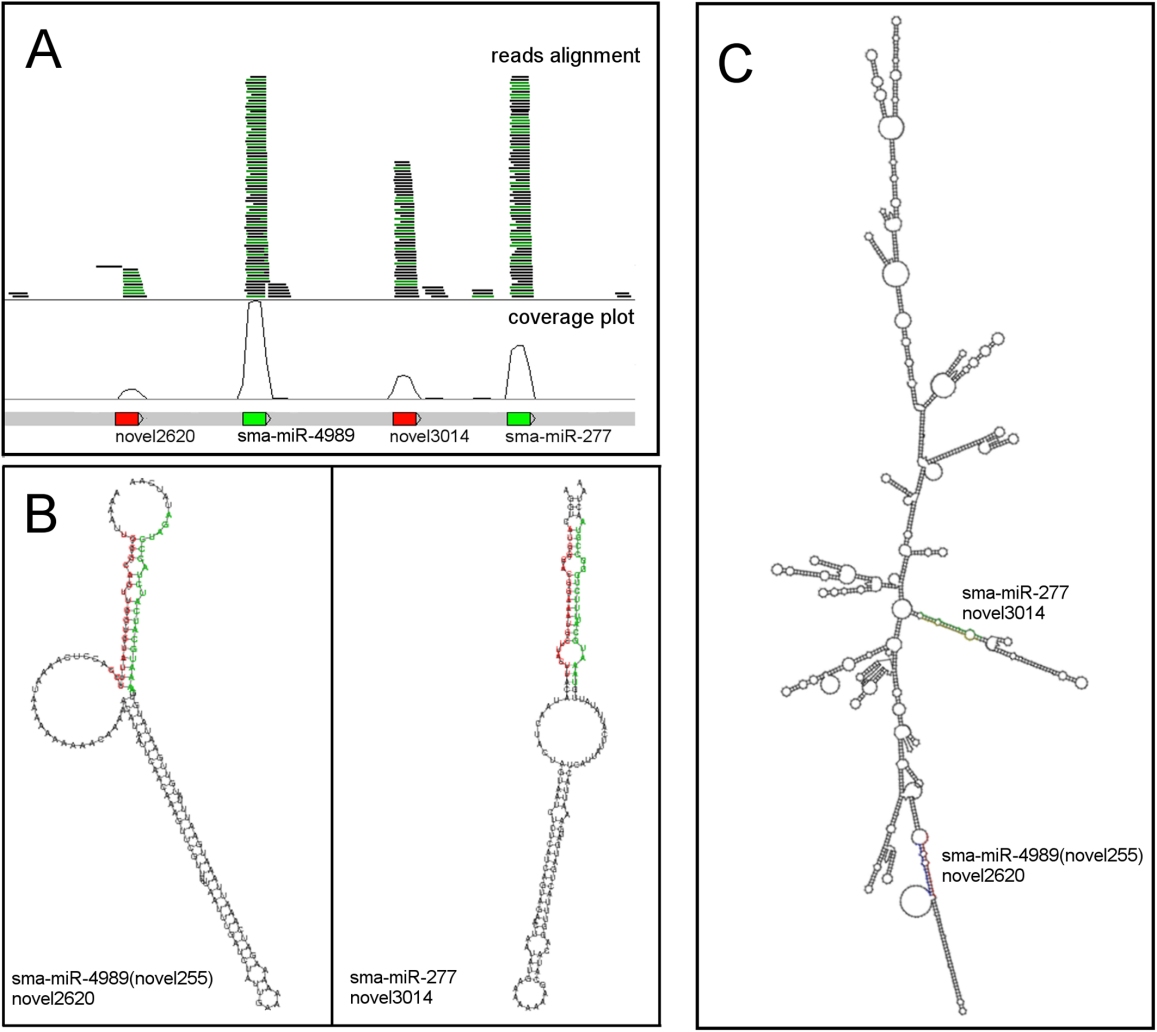
Sma-miR-277 and sma-miR-4989 belong to a gene cluster. (A) The genomic locus in Chromosome 4 of sma-miR-277 and sma-miR-4989 suggests they belong to a gene cluster. The average distance between genes (represented by coloured boxes) is 109 bases. Here the mature miRNAs (sma-miR2-277 and sma-miR-4989) and passenger miRNAs are represented with coverage plot and aligned reads from one of the small RNA libraries. (B) Predicted stem-loop structures for sma-miR-277 and sma-miR-4989 –individual cases. Mature miRNAs are located in the 3’-end of the hairpin. (C) Due to the cluster organisation of sma-miR-277/sma-miR-4989, it is likely that they are transcribed as one precursor RNA molecule. This figure represents the predicted stem-loop structure for sma-miR-277/sma-miR-4989 when arising from a larger transcript.

Sma-miR-277 has previously been found in vesicles derived from cultured larval stages of *S*. *mansoni* [75] as well as circulating in naturally infected patients and experimentally infected animals [76]. However, in both reports the genomic location of this miRNA was not described. In tapeworms, miR-277 is located within a cluster with miR-4989 [77], i.e. they are very close together in the genome (less that 10 Kb) and miRNAs encoded in clusters are likely to be transcribed as a single precursor unit [78]. A search for the genome locus of miR-277 in *S*. *mansoni* showed that it is found in Chromosome 4 (21600667–21600688—reverse). We extended our analysis to include alignments of previously generated small RNA-seq libraries resulting in the identification of another miR-277 family member located in the vicinity of sma-miR-277. Sma-miR-4989 (named by us “novel255”) is located within ~200 bp of sma-miR-277 suggesting that, as is the case in tapeworms, these two miRNAs form a miRNA cluster. Other sequencing reads found in the same region suggested the presence of passenger strands or miRNA* (Fig 2A). We found that sma-miR-4989 (novel255) folds into a stable structure with its passenger strand (novel2620), the two miRNAs matching to form the lower part in a precursor with an extended stem. The miRNAs sma-miR-277 and its passenger (novel3014) match up similarly, again forming the lower part of an extended stem (Fig 2B). These two extended stem-loop structures were consistently identified among different excisions of the region. They persist when folding the whole (1,700 nt) genomic loci (Fig 2C), where the region folds into a highly stable stem-rich structure with low minimum free energy. To assess the significance of this fold, we randomly shuffled its sequence while preserving dinucleotide frequency. An ensemble of 1,000 randomly shuffled sequences was folded. This yielded a distribution of minimum free energy scores, leading to a Z-score of 3.5 for minimum free energy of the genomic sequence fold, confirming the stability of the stem-rich structure. FASTA sequences for the hairpins, mature and passenger strands are presented in Supplementary S2 Dataset and an image comparing the folds obtained for different *S*. *mansoni* predicted miRNAs is presented in Supplementary S4 Fig.

### 2.3. Targets of sma-miR-277/sma-miR-4989 are down regulated in paired females compared with unpaired females

**Fig 3:**
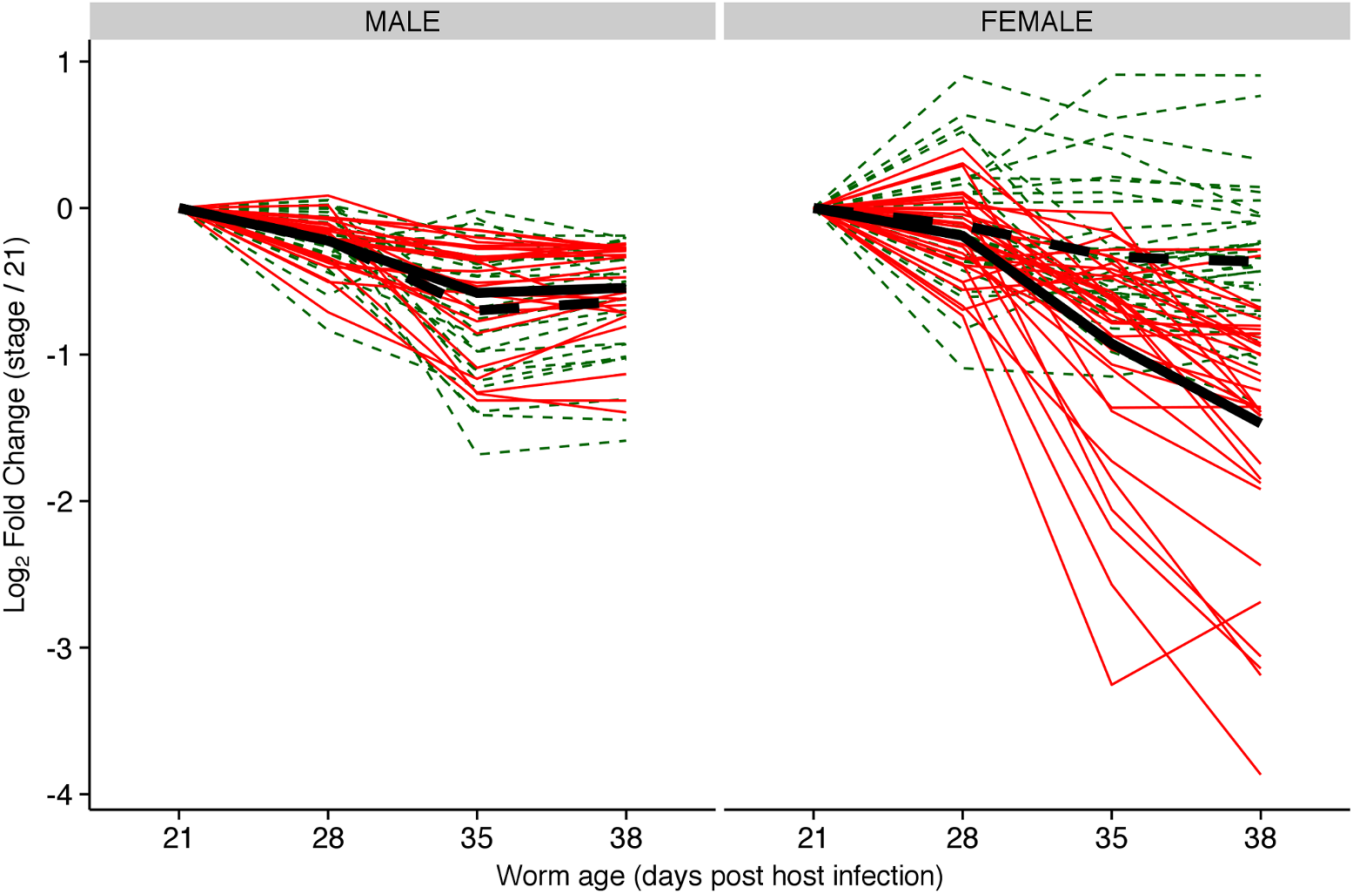
Sma-miR-277 family predicted targets downregulated in developing female parasites. Fold change expression (Log_2_) of high confidence targets of sma-miR-277 family during the development of male and female worms in two conditions: paired (solid line red) and unpaired (dashed green). Black lines represent the mean expression of genes in paired (solid black line) and unpaired (dashed black line) worms.

Female worms only achieve sexual maturity when they are coupled with a male worm. Although highly unlikely in natural infections, it is possible to obtain unpaired “virgin” females and unpaired males by infecting mice with single-sex cercariae. Using RNASeq data from paired and unpaired males and females, we found that 16 out of the 34-female high-confidence targets of sma-miR-277 family had significantly (adjusted p-value <0.01) lower expression in paired females compared to unpaired “virgin” females (Fig 3B), while the male targets were not differentially expressed between paired and unpaired males (Fig 3A). Furthermore, the time-course expression analysis indicated that the expression of these targets was gradually reduced as female maturation progressed. Notably, this was not observed in males.

### 2.4. Sma-miR-4989 is up-regulated during juvenile to adult transition

Tentative miR-277 targets were down-regulated during the juvenile to adult transition. If the miRNA effect is that of causing the decay of its targets, it is expected that the expression of miRNAs from that family would show an opposite pattern to that of its targets, i.e. while the targets of the miR-277 family are down-regulated, sma-miR-277 and sma-miR-4989 should increase their expression.

**Fig 4:**
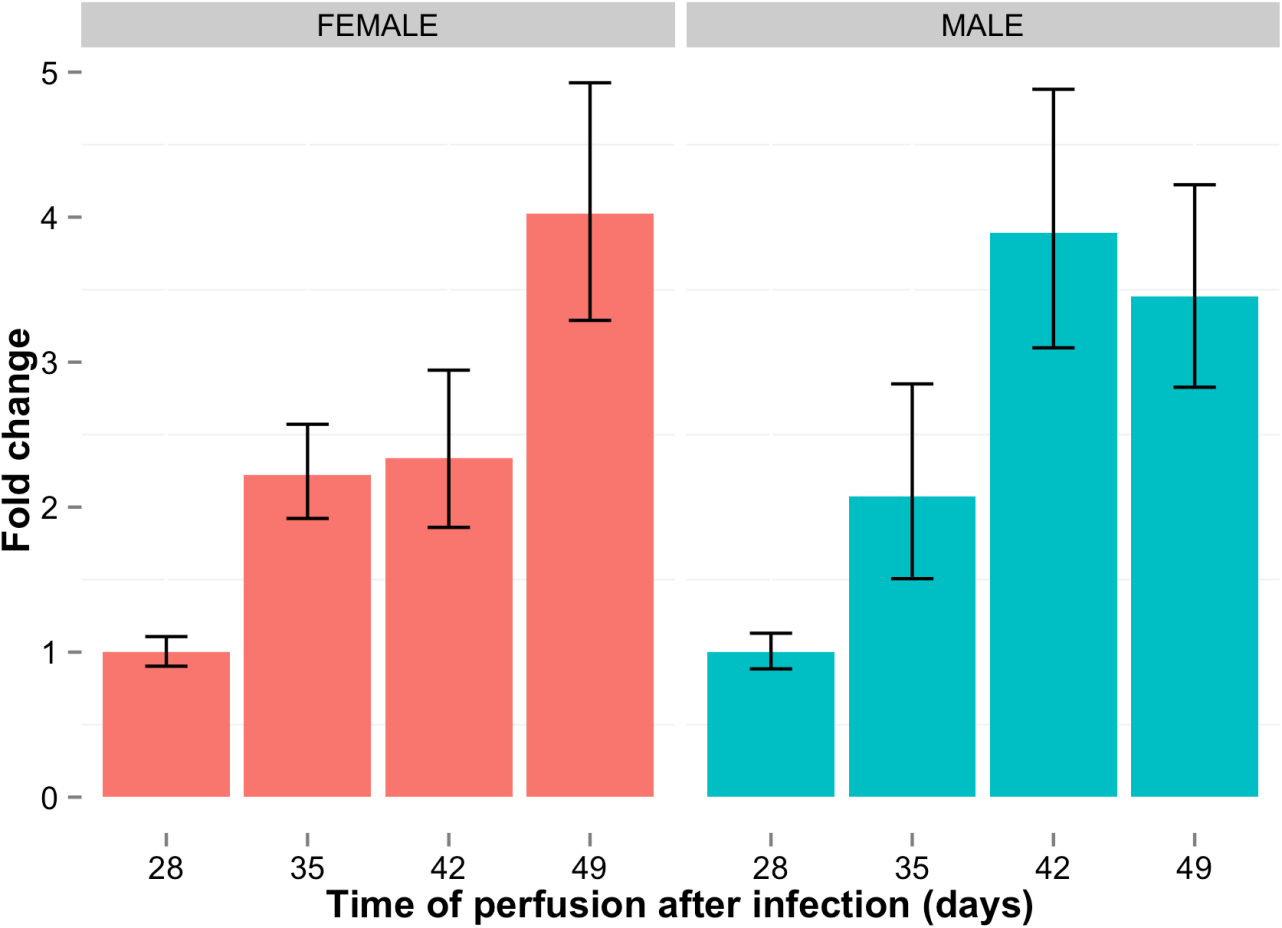
Sma-miR-4989 is significantly up-regulated during male and female maturation. Fold change expression of sma-miR-4989 during development of juvenile to adult worms in male (blue bars) and females (red bars) as measured by RT-qPCR. Samples were collected at the time points (days post infection) indicated in the x-axis from murine hosts infected with pooled (mixed sex) cercariae. Each barplot represents the mean of three biological replicates. T-tests were performed between 49 d.p.i. and 28 d.p.i. and were both significant with p-value ≤ 0.01. Error bars show the standard error of the mean, based on three biological replicates.

The expression sma-miR-4989 was quantified by RT-qPCR during juvenile to adult development. This miRNA showed significant up-regulated (28 vs. 49 d.p.i., males t-test p-value = 0.0097; females t-test p-value = 0.01) expression in developing worms (Fig 4) suggesting an association between increased expression in miRNA levels and decreased expression of their targets. Furthermore, sma-miR-277 is also up-regulated in a similar manner, as well as sma-miR-4989 passenger miRNA novel2620 (Supplementary S3 Fig).

### 2.5. Sma-miR-4989 mRNA is localised in the male anterior oesophagus gland

**Fig 5:**
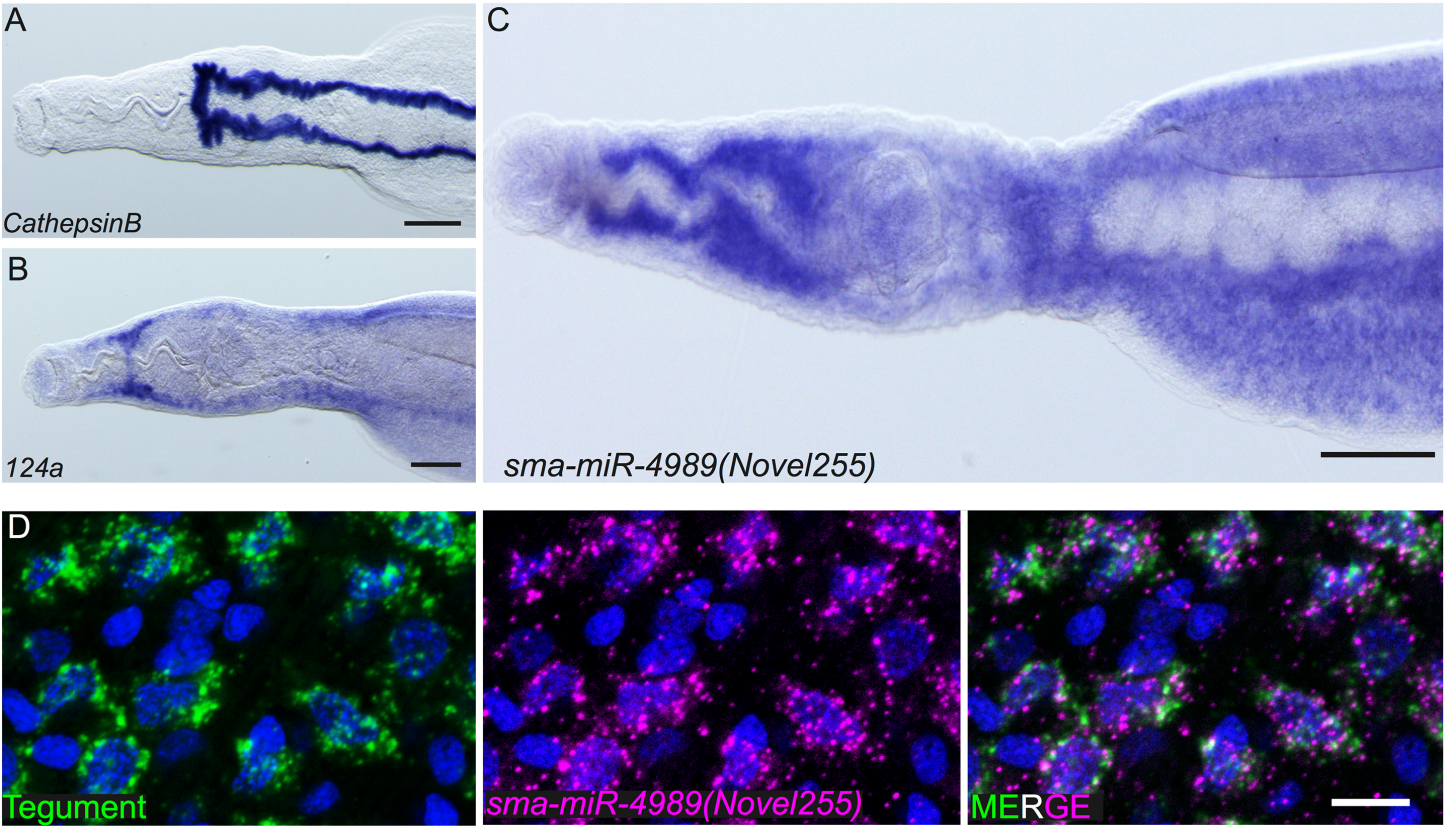
Sma-miR-4989 is expressed in the cells surrounding the oesophagus and cells of the tegument in adult worms. Whole mount *in situ* hybridisation for (A) *cathepsin B*, (B) sma-miR-124a-3p (*124a*), and (C) sma-miR-4989. (D) Fluorescence *in situ* hybridisation showing the colocalization of sma-miR-4989 with four co-expressed tegument-specific mRNAs (*calpain*, *npp-5*, *annexin* and *gtp-4*). Nuclei are stained with DAPI and shown in blue. Anterior of worms is to the left in A-C. Scale Bars: A-C 100 μm; D 10 μm.

In order to gain insight into the potential functions of sma-miR-4989, we used both whole mount *in situ* hybridisation (WISH), as well as fluorescence *in situ* hybridization (FISH) in adult male and female worms. Given the small size of miRNAs we employed anti-sense Locked Nucleic Acid (LNA) probes to detect the cells expressing these RNAs. To determine the specificity of this approach we performed control experiments with LNA probes targeting a 22nt long sequence of the *CathepsinB* messenger RNA (Smp_103610), which is detected in the intestine by WISH [68,79]. Consistent with previous reports, we robustly detected *cathepsinB* in the intestine (Fig 5A). To further assess the feasibility to detect Schistosome miRNAs using LNA probes we also examined sma-miR-124a-3p, whose closely related *C*. *elegans* homolog (miR-124a) is expressed in the nervous system [80]. Consistent with the localization of miR-124a in *C*. *elegans*, we detected the expression of sma-miR-124a-3p in the schistosome cephalic ganglia and in the nerve chords by WISH (Fig 5B). To determine if this miRNA was broadly expressed in the cephalic ganglia or in subsets of cells in the cephalic ganglia, we performed double FISH with *prohormone convertase 2* (*pc2*), that is expressed in the large number of cells in the schistosome nervous system. Schistosomes possess a pair of cephalic ganglia whose neuronal cell bodies surround a neuropil composed of a network of neural projections. The individual ganglia are connected via a commissure of neural projections that extend across the midline. We observed that sma-miR-124a-3p and *pc2* were co-expressed in cell bodies that comprise both the cephalic ganglia and the nerve chords (Supplementary S5A Fig). Interestingly, we detected sma-miR-124a-3p not just in pc2^+^ cell bodies but also broadly throughout the neuropil of the cephalic ganglia (Supplementary S5B Fig). Since the neuropil is comprised of neural projections (axons and dendrites) these data suggest that sma-miR-124a-3p may have functions to regulate its target mRNAs outside the cell bodies.

Given our success in detecting sma-miR-124a-3p, we set out to determine the localization of sma-miR-4989. By WISH, we failed to detect reproducible signal in female parasites. However, we strongly detected sma-miR-4989 in cells surrounding the male oesophagus (Fig 5C) as well as cells throughout in the parenchyma. Since many of the sma-miR-4989-expressing cells in the parenchyma appeared to be quite superficial, just beneath the parasites muscle layer, we explored the possibility these cells comprise the schistosome syncytial epidermis, a structure known as the tegument. To test this, we utilized a mix of probes targeting the mRNAs of four well-characterized tegumental factors: *calpain*, *npp-5*, *annexin* and *gtp-4* (ref. [69–73]). By FISH we observed that the sma-miR-4989 RNA was broadly expressed in cells that make up the schistosome tegument (Fig 5D).

## Discussion

In this study, the contribution of miRNAs in shaping the transcriptional landscape of developing *S*. *mansoni* juveniles was evaluated by performing a Sylamer analysis [36]. This approach is independent from any prior miRNA information available, i.e. miRNA-unaware. Using Sylamer we were able to show that transcripts containing a target site for members of the miR-277 family are highly enriched in early juvenile worms and their expression dramatically decreases towards adulthood (Fig 1A). Remarkably, no other known miRNA seeds were found enriched in this analysis, suggesting that members of the miR-277 family may be the primary miRNAs exerting post-transcriptional regulation in the transition from juvenile to adult worm.

Sylamer results were then coupled with a stringent miRNA target prediction approach that uses miRanda [54] and TargetScan with species conservation [55]. The latter algorithm allows the inclusion of evolutionarily conserved miRNA targets from the three main schistosoma species. The rather low overlap between the Sylamer miRNA-unaware approach and the miRNA-driven TargetScan with conservation and miRanda (12+14+20 targets, Fig 1B) would not represent a disagreement between methods; on the contrary, they complement each other. While the combination of TargetScan with conservation and miRanda provides a highly confident list of miRNA targets, Sylamer provides a statistical approach to the identification of transcripts potentially targeted by a given miRNA based on their co-regulated change in expression. This combined approach, which includes the aforementioned unaware miRNA-target finder component, has never before been applied to schistosomes and our results provide solid evidence for the role of miRNAs during the intra-mammalian development of this parasite. We conclude that the overlap among these methods does identify a group of genes regulated by members of the miR-277. Further *in silico* functional characterisation of the targets showed that the miR-277 family might be responsible for the down-regulation of important transcription factors, transcription factors associated proteins and signalling molecules (Table 1), for instance a TATA binding box protein associated factor, Tbox transcription factor, a homolog of the *S*. *mediterranea* p53 protein known to regulate proliferation and self-renewal in stem-cells [81] and a groucho domain-containing protein. The *Drosophila* Groucho (Gro) protein is a corepressor whose action is required, among other processes, for sexual determination [82]. In schistosomes, the 3’-UTR of the gene encoding groucho (Smp_165280) has three miRNA target recognition sites suggesting close regulation by sma-miR-277/4989 (Supplementary S6 Table). Interestingly, one of the three Argonaute proteins known to be encoded in the *S*. *mansoni* genome [83] is found among the miR-277 targets. Argonaute is part of the RNA–induced silencing complex (RISC), a key player in the RNA interference pathway [84] and the potential targeting of Argonaute by a miRNAs could suggest a possible feedback loop between the expression of miRNAs and the RNAi effector pathway.

Sixteen of the high confidence targets (potential miR-277 targets that are down-regulated towards adulthood) are also differentially expressed in “virgin” unpaired females compared to sexually active mature females, suggesting that miR-277 family members are involved in reproductive development. Other miRNAs have recently been linked to reproductive development in *S*. *japonicum* [32]. The post-transcriptional regulation of key players during the transition between developmental stages is consistent with the role predicted for certain miRNAs in other organisms (reviewed in [35]).

The miR-277 family is of particular interest in that, to date, it has only been found in protostomes [85]. In *Schmidtea mediterranea*, a model free-living flatworm for the study of tissue regeneration, Sasidharan *et al*. identified four members of the miR-277 family [86]. In three cestodes (*E*. *granulosus*, *E*. *multilocularis* and *Hymenolepis microstoma*), the gene loci encoding miR-277 and miR-4989 (another member of the miR-277 family) are ~ 430 bases apart, comprising a miRNA cluster [87] with gene expression potentially co-regulated. In schistosomes, sma-miR-277 has been detected in sera from experimentally-infected animals and humans naturally infected with schistosomes [76] as well as in secreted vesicles of schistosomula larvae [75]. A recent publication featuring miRNA profiling in developing *S*. *japonicum* worms identified both sja-miR-277 and sja-miR-277b [32], the latter being identical to our sma-miR-4989. Our results suggest that sma-miR-4989 is the *S*. *mansoni* homolog of sja-miR-277b.

The high quality of the *S*. *mansoni* genome assembly allowed us to localise both sma-miR-277 and sma-miR-4989 within a locus containing clustered miRNA genes (Fig 2A) in Chromosome 4. Given the conserved architecture of this miRNA genomic locus when compared to that of tapeworms, and the sequence conservation, we have therefore named novel255 as sma-miR-4989. The miRNA precursor structure results for sma-miR-4989 and sma-miR-277 were found to require longer flanking regions to achieve a thermodynamically stable stem-loop structure (Fig 2B and Fig 2C). This structural difference found in these *Schistosoma* miRNA precursors (Supplementary S4B Fig and Supplementary S4C Fig), when compared to canonical structures (i.e. Supplementary S4A Fig) described for model organisms, could potentially explain previous claims that flatworms might have lost many of the conserved miRNA families [85]. In addition, the highly fragmented nature of the current *S*. *japonicum* [88] and previous *S*. *mansoni* genome assemblies [89] may explain the difficulties previously encountered in identifying these particular miRNAs.

Regarding miRNA expression, our results show that sma-miR-4989 displays the same pattern of expression in male and female worms during development (Fig 4). These results are in agreement with recently published work which showed that *S*. *japonicum* sja-miR-277 and sja-miR-277b (homolog to sma-miR-4989) are both differentially expressed in the transition from juvenile to adult as well as in between male and female *S*. *japonicum* worms [32]. Given that the expression pattern of sma-miR-277/4989 and their predicted targets show an inverse correlation, we speculate that the post-transcriptional effect of these miRNAs might be exerted by degradation of the target mRNA [34].

As part of our functional characterisation of sma-miR-4989, we performed whole-mount *in situ* hybridisation (WISH) experiments to localise the site of expression of this miRNA in the worms. Our results show that sma-miR-4898 is expressed in tegumental cells along the whole of the male worm body but its expression is most pronounced in the cells surrounding oesophagus in proximity to the oesophageal glands [90,91]. Detailed transmission electron microscopy of the oesophageal region has shown that the schistosome oesophagus has two distinct regions [92]: the posterior region that is involved in initialling the blood meal while the cellular structure of the anterior region has been described as similar to the tegument, with cell bodies located beneath the muscle fibres and projecting cytoplasmatic extensions that end in the oesophageal lumen. The discoid bodies and multilaminate vesicles that are exported to the oesophagus lumen reveal the glandular nature of the anterior oesophagus and suggest that their secretions might constitute the building blocks of the membranous lining of the oesophagus [90,91,93]. At present, it is difficult to determine if sma-miR-4898 is expressed in these oesophageal glands or in the tegument-like tissue that lines the oesophagus. Although RNA contents of adult oesophagus gland secretions have yet to be analysed in detail, a study of the *S*. *mansoni* schistosomula vesicles and exosomes [75] identified several known and novel miRNAs, including sma-miR-277. Given the localisation of sma-miR-4989 in the tegumental cells, we speculate that these molecules could be reaching the exterior of the worm in vesicle-like structures. Further work is needed to test this hypothesis.

In summary, the high quality nature of the *S*. *mansoni* genome and gene annotations allowed us to query 3’-UTR regions for miRNAs target recognition sites that are conserved across three Schistosoma species. By incorporating gene expression information, we were able to conclude that that sma-miR-277 and sma-miR-4989 may be the primordial miRNAs driving an effect on the transcriptional landscape regulating gene expression changes underlying the development of juveniles and sexual maturation. Further, we showed that sma-miR-277 and sma-miR-4989 expression is increased towards adulthood, this is consistent with evidence that their targets are down-regulated during the same transition. Finally, we report the first use of a standard WISH technique to the localisation of miRNAs in schistosomes, demonstrating that sma-miR4989 is expressed in tegumental cells and raising many questions about the potential roles of this miRNA.

## Supporting information

S1 Fig Reverse transcription PCR reactions for 12 UTRs. Lanes from left to right: Ladder; 1, 2: Smp_152790.1; 3, 4: Smp_149640.1, 5, 6: Smp_147920.1; 7, 8: Smp_144140.1; 9, 10: Smp_200240.1; 11, 12: Smp_159780.1; 13, 14: Smp_186020.1; 15, 16: Smp_159570.1; 17, 18: Smp_133210.1; 19, 20: Smp_213910.1; 21, 22: Smp_154340.1; 23–30: empty; ladder; positive control. Odd numbers represent the test PCR while even numbers represent the “-RT” (without reverse transcriptase) control.
(DOCX)

S2 Fig Distribution of targets (mRNA) of sma-miR-277 miRNA family across the expression spectrum of all transcripts in adult worms. Sylamer and high confidence targets of sma-miR-4989(novel255) are spread across the differential expression ranking set of genes. Each point represents a transcript that is significantly higher expressed in juveniles. Highlighted in orange are those transcripts that, in addition, contained a sma-miR-277 miRNA family target in their UTR according to Sylamer. Blue dots represent the subset of Sylamer genes whose orthologs in *S*. *haematobium* and *S*. *japonicum* also have a sma-miR-277 miRNA family target. These are regarded as high confidence targets as they are confirmed independently by three methods (i.e. Sylamer, miRanda and TargetScan with conservation).
(DOCX)

S3 Fig Expression of selected miRNAs during juvenile to adult development. Fold change expression of sma-miR-277, novel2620 and sma-miR-4989(novel255) during development of juvenile to adult worms in male (blue bars) and females (red bars) as measured by RT-qPCR. Samples were collected at the time points (days post infection) indicated in the x-axis from murine hosts infected with pooled (mixed sex) cercariae. In the case of sma-miR-4989(novel255) these data were independently collected from that shown in Fig 4 of the main text. Error bars represent the standard error of the mean, based on three biological replicates.
(DOCX)

S4 Fig Stem-loop structures predicted for known and novel small RNAs in *Schistosoma mansoni*. (A) Examples of “canonical” hairpins. (B) and (C) Examples of hairpins that required longer flanking regions to achieve a thermodynamically stable stem-loop structure. Part (C) depicts those with a characteristic side “bulge”.
(DOCX)

S5 Fig Fluorescence *in situ* hybridization of sma-miR-124. Fluorescence *in situ* hybridization (FISH) in adult male worms using a Locked Nucleic Acid (LNA) probe to detect sma-miR-124a-3p (instead of antisense mRNA probes) and *prohormone convertase 2* (*pc2*), that is expressed in the large number of cells in the schistosome nervous system. (A) shows detection of the expression of sma-miR-124a-3p and *pc2* in the schistosome cephalic ganglia and in the nerve chords. (B) zoomed-in view of (A) showing the presence of sma-miR-124a-3p in the neural projections that connect the two cephalic ganglia (indicated with arrows) while *pc2* is restricted to the cell bodies in the cephalic ganglia.
(DOCX)

S1 Table List of primers (gene id and sequence) used for UTR validation. (XLS)

S2 Table Kraken output from small RNA sequencing processing. (XLS)

S3 Table Sylamer table output listing identified 7-mers and their respective p-value. (XLS)

S4 Table List of miRNA targets found using MiRanda and TargetScan with conservation in the three Schistosoma species (*S*. *mansoni*, *S*. *japonicum* and *S*. *haematobuim*). (XLS)

S5 Table Gene Ontology (GO) term enrichment (Biological processes only) of high confidence sma-miR-277 and sma-miR-4989(novel255) targets. (XLS)

S6 Table Location of sma-miR-4989(novel255) target sites (SCR) in *S*. *mansoni* transcripts. (XLS)

S1 Text RNA isolation using phase extraction and ETOH precipitation–suitable for samples with high carbohydrate content. (DOCX)

S1 Dataset GTF (annotation) file used for transcriptome analysis. (ZIP)

S2 Dataset FASTA sequences for the hairpins, mature and passanger strands. (TXT)
