# Correction: MiR-277/4989 regulate transcriptional landscape during juvenile to adult transition in the parasitic helminth *Schistosoma mansoni*

**DOI:** 10.1371/journal.pntd.0007121

**Published:** 2022-06-02

**Authors:** The PLOS Neglected Tropical Diseases Staff

There are errors in the Supporting Information. S1 Text is the incorrect file, and S2 Text has been omitted from the list of Supporting Information. The correct S1 and S2 Text can be viewed below. The publisher apologizes for the errors.

## Supporting information

S1 Text Protocol. RNA isolation using phase extraction and ETOH precipitation—suitable for samples with high carbohydrate content.
(DOCX)

S2 Text List of Taqman rt-qPCR miRNA assays. (PDF)
