# Correction: MiR-277/4989 regulate transcriptional landscape during juvenile to adult transition in the parasitic helminth *Schistosoma mansoni*

**DOI:** 10.1371/journal.pntd.0010521

**Published:** 2022-06-06

**Authors:** Anna V. Protasio, Stijn van Dongen, Julie Collins, Leonor Quintais, Diogo M. Ribeiro, Florian Sessler, Martin Hunt, Gabriel Rinaldi, James J. Collins, Anton J. Enright, Matthew Berriman

There is an error in the legend for S1 Fig. Please see the correct legend and S1 Fig below.

S2 Text was incorrectly published with the legend for S1 Text, while the correct S1 Text was not published. Please see the correct S1 and S2 Text files below.

Raw data files from qPCR output underlying Fig 4 and S3 Fig were not published. The author has provided these files as S3–S11 Dataset.

Sample details of the RNAseq data under accession E-ERAD-478 have been provided as S11 Dataset.

The Data Availability Statement is incomplete. The Data Availabilty Statement should read: Schistosomula poly-A enriched and small RNAseq data: European Nucleotide Archive study accession number PRJEB3190. Adult poly-A enriched RNAseq data: ArrayExpress accession number E-ERAD-478. Code used to parse data, calculate RQ, plot and produce the t-test are available at https://github.com/annaprotasio/Protasio_et_al_2017_plosNTD. All other data are included in the manuscript and Supporting Information files.

## Supporting information

S1 Fig Reverse transcription PCR reactions for 12 UTRs. Odd numbers represent the test PCR while even numbers represent the “-RT” (without reverse transcriptase) control. PCR control is done against UTR of alpha tubulin Smp_090120.1.
(PDF)

S1 Text Protocol. RNA isolation using phase extraction and ETOH precipitation—suitable for samples with high carbohydrate content.
(DOCX)

S2 Text List of Taqman rt-qPCR miRNA assays. (DOCX)

S3 Dataset Raw qPCR data for females 28 d.p.i. (XLS)

S4 Dataset Raw qPCR data for females 35 d.p.i (XLS)

S5 Dataset Raw qPCR data for females 42 d.p.i. (XLS)

S6 Dataset Raw qPCR data for females 49 d.p.i. (XLS)

S7 Dataset Raw qPCR data for males 28 d.p.i. (XLS)

S8 Dataset Raw qPCR data for males 35 d.p.i. (XLS)

S9 Dataset Raw qPCR data for males 42 d.p.i. (XLS)

S10 Dataset Raw qPCR data for males 49 d.p.i. (XLS)

S11 Dataset RNAseq data details for accession E-ERAD-478. (XLSX)
