# Supplementary material for: MiR-277/4989 regulate transcriptional landscape during juvenile to adult transition in the parasitic helminth Schistosoma mansoni
Source: PLoS Negl Trop Dis. 2017 May 23;11(5):e0005559. doi: 10.1371/journal.pntd.0005559 (PMC5459504; doi:10.1371/journal.pntd.0005559)
Supplement: S1 Fig — Lanes from left to right: Ladder; 1, 2: Smp_152790.1; 3, 4: Smp_149640.1, 5, 6: Smp_147920.1; 7, 8: Smp_144140.1; 9, 10: Smp_200240.1; 11, 12: Smp_159780.1; 13, 14: Smp_186020.1; 15, 16: Smp_159570.1; 17, 18: Smp_133210.1; 19, 20: Smp_213910.1; 21, 22: Smp_154340.1; 23–30: empty; ladder; positive control. Odd numbers represent the test PCR while even numbers represent the “-RT” (without reverse transcriptase) control. (DOCX) [file pntd.0005559.s001.docx]

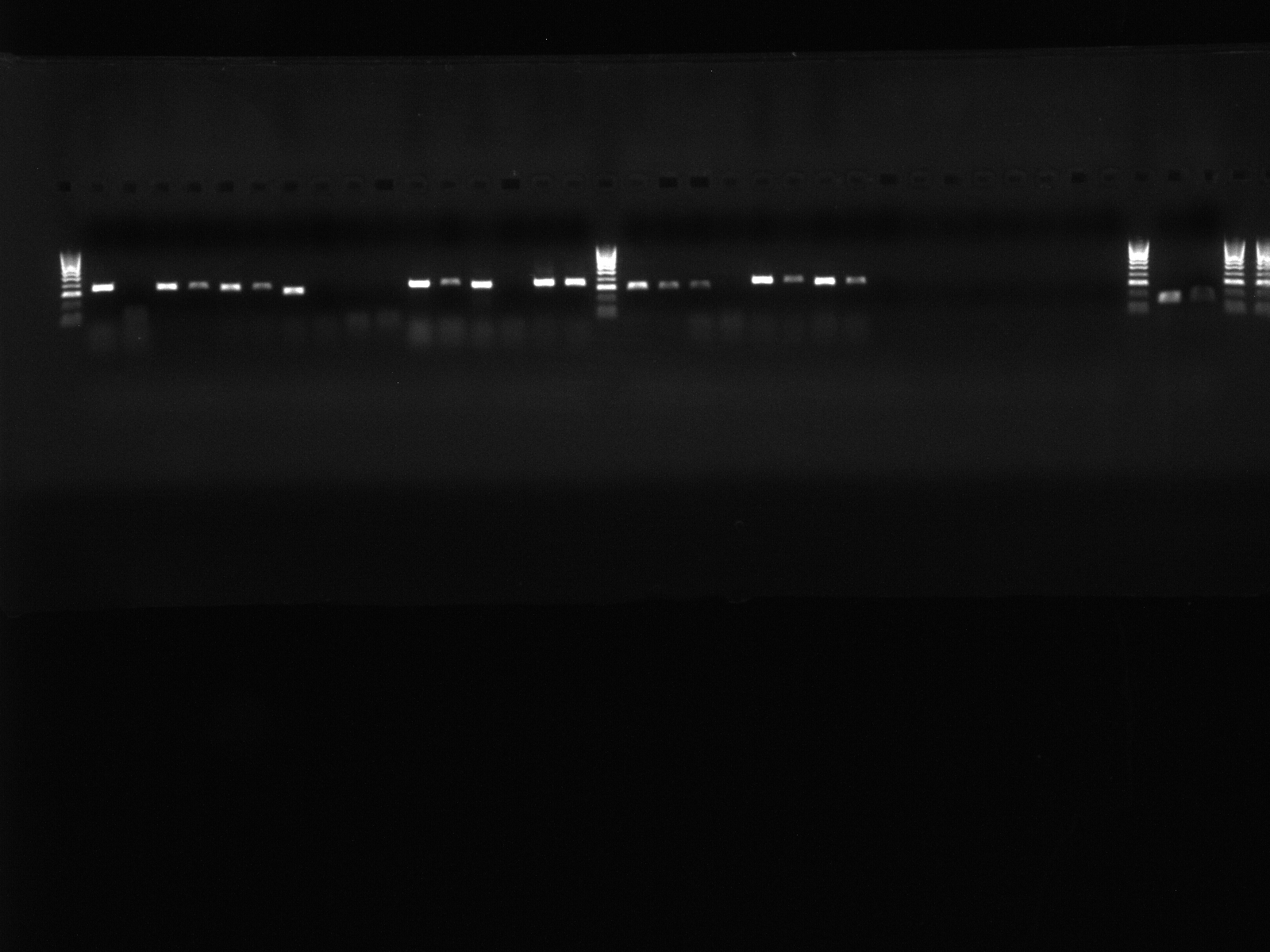


**Supplementary Figure S1. Reverse transcription PCR reactions for 12 UTRs.**

Lanes from left to right: Ladder; 1, 2: Smp_152790.1; 3, 4: Smp_149640.1, 5, 6: Smp_147920.1; 7, 8: Smp_144140.1; 9, 10: Smp_200240.1; 11, 12: Smp_159780.1; 13, 14: Smp_186020.1; 15, 16: Smp_159570.1; 17, 18: Smp_133210.1; 19, 20: Smp_213910.1; 21, 22: Smp_154340.1; 23-30: empty; ladder; positive control. Odd numbers represent the test PCR while even numbers represent the “-RT” (without reverse transcriptase) control.
