# Supplementary material for: MiR-277/4989 regulate transcriptional landscape during juvenile to adult transition in the parasitic helminth Schistosoma mansoni
Source: PLoS Negl Trop Dis. 2017 May 23;11(5):e0005559. doi: 10.1371/journal.pntd.0005559 (PMC5459504; doi:10.1371/journal.pntd.0005559)
Supplement: S2 Fig — Sylamer and high confidence targets of sma-miR-4989(novel255) are spread across the differential expression ranking set of genes. Each point represents a transcript that is significantly higher expressed in juveniles. Highlighted in orange are those transcripts that, in addition, contained a sma-miR-277 miRNA family target in their UTR according to Sylamer. Blue dots represent the subset of Sylamer genes whose orthologs in S. haematobium and S. japonicum also have a sma-miR-277 miRNA family target. These are regarded as high confidence targets as they are confirmed independently by three methods (i.e. Sylamer, miRanda and TargetScan with conservation). (DOCX) [file pntd.0005559.s002.docx]

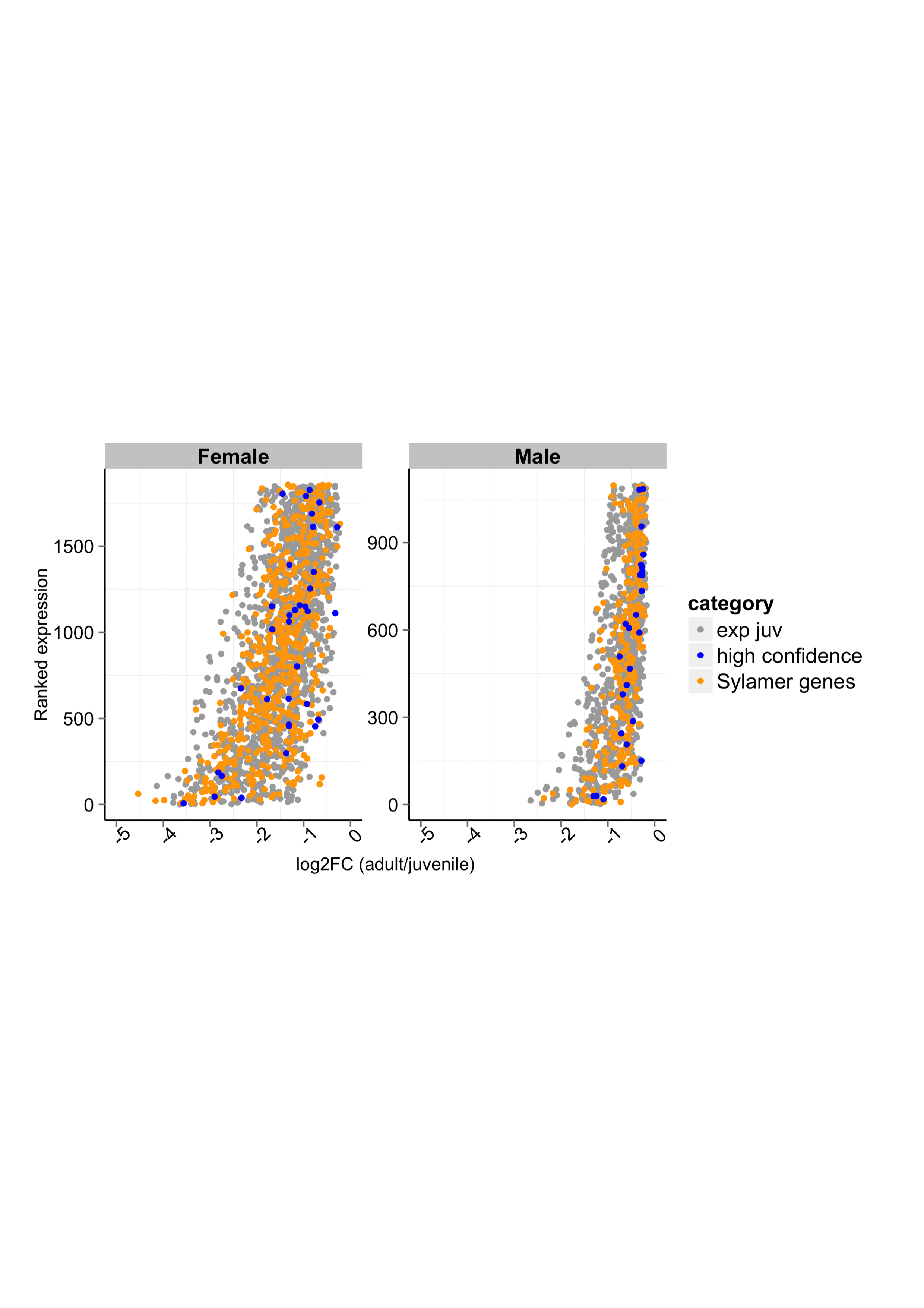


**Supplementary figure 2. Distribution of** **targets (mRNA) of** **sma-miR-277 miRNA family across the expression spectrum of all transcripts in adult worms.**

Sylamer and high confidence targets of sma-miR-4989(novel255) are spread across the differential expression ranking set of genes. Each point represents a transcript that is significantly higher expressed in juveniles. Highlighted in orange are those transcripts that, in addition, contained a sma-miR-277 miRNA family target in their UTR according to Sylamer. Blue dots represent the subset of Sylamer genes whose orthologs in *S. haematobium* and *S. japonicum* also have a sma-miR-277 miRNA family target. These are regarded as high confidence targets as they are confirmed independently by three methods (i.e. Sylamer, miRanda and TargetScan with conservation).
