# Supplementary material for: MiR-277/4989 regulate transcriptional landscape during juvenile to adult transition in the parasitic helminth Schistosoma mansoni
Source: PLoS Negl Trop Dis. 2017 May 23;11(5):e0005559. doi: 10.1371/journal.pntd.0005559 (PMC5459504; doi:10.1371/journal.pntd.0005559)
Supplement: S3 Fig — Fold change expression of sma-miR-277, novel2620 and sma-miR-4989(novel255) during development of juvenile to adult worms in male (blue bars) and females (red bars) as measured by RT-qPCR. Samples were collected at the time points (days post infection) indicated in the x-axis from murine hosts infected with pooled (mixed sex) cercariae. In the case of sma-miR-4989(novel255) these data were independently collected from that shown in Fig 4 of the main text. Error bars represent the standard error of the mean, based on three biological replicates. (DOCX) [file pntd.0005559.s003.docx]

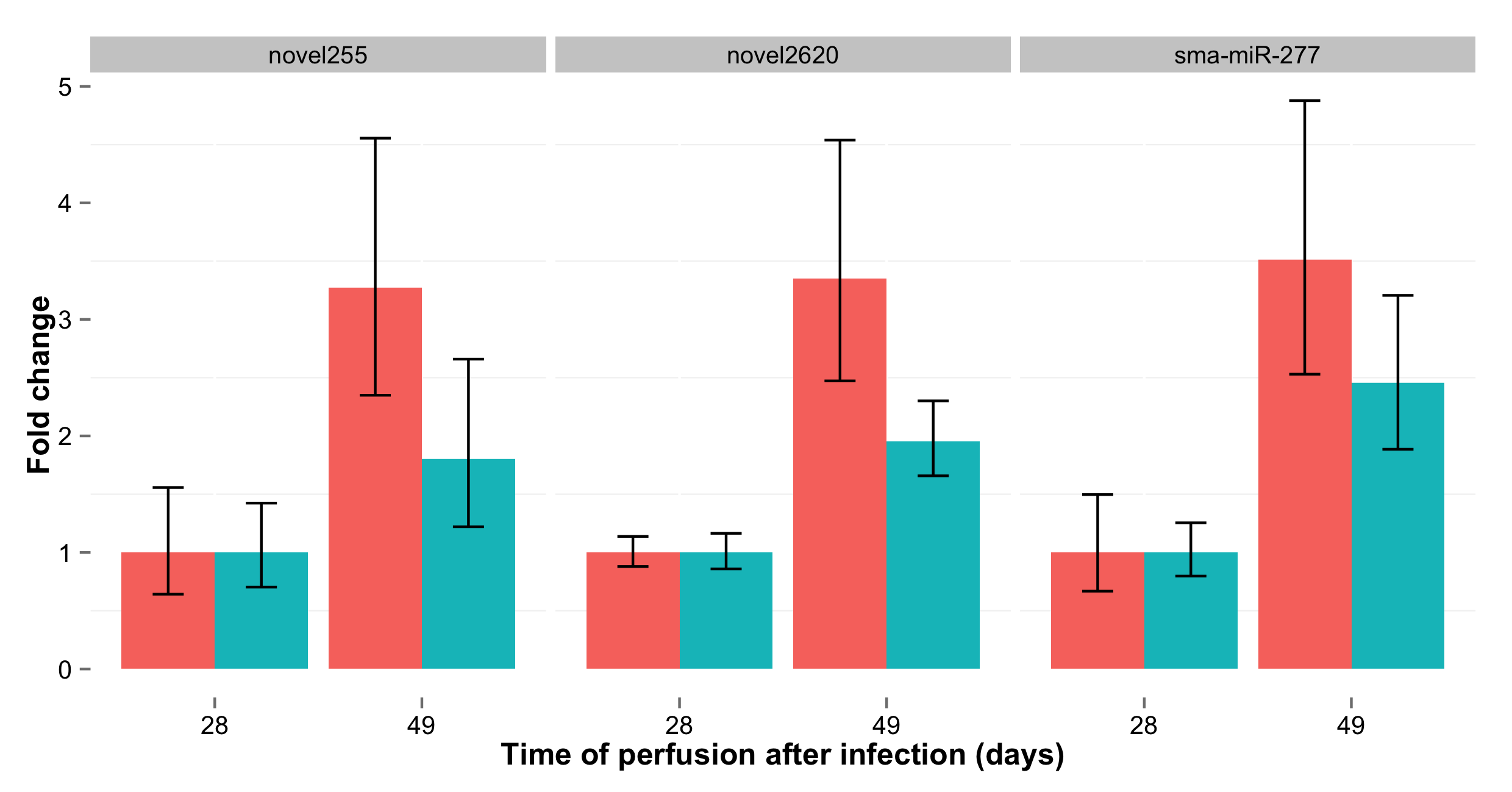


**Supplementary Figure 3. Expression of selected miRNAs during juvenile to adult development.**

Fold change expression of sma-miR-277, novel2620 and sma-miR-4989(novel255) during development of juvenile to adult worms in male (blue bars) and females (red bars) as measured by RT-qPCR. Samples were collected at the time points (days post infection) indicated in the x-axis from murine hosts infected with pooled (mixed sex) cercariae. In the case of sma-miR-4989(novel255) these data were independently collected from that shown in Figure 4 of the main text. Error bars represent the standard error of the mean, based on three biological replicates.
