# Supplementary material for: MiR-277/4989 regulate transcriptional landscape during juvenile to adult transition in the parasitic helminth Schistosoma mansoni
Source: PLoS Negl Trop Dis. 2017 May 23;11(5):e0005559. doi: 10.1371/journal.pntd.0005559 (PMC5459504; doi:10.1371/journal.pntd.0005559)
Supplement: S4 Fig — (A) Examples of “canonical” hairpins. (B) and (C) Examples of hairpins that required longer flanking regions to achieve a thermodynamically stable stem-loop structure. Part (C) depicts those with a characteristic side “bulge”. (DOCX) [file pntd.0005559.s004.docx]

**
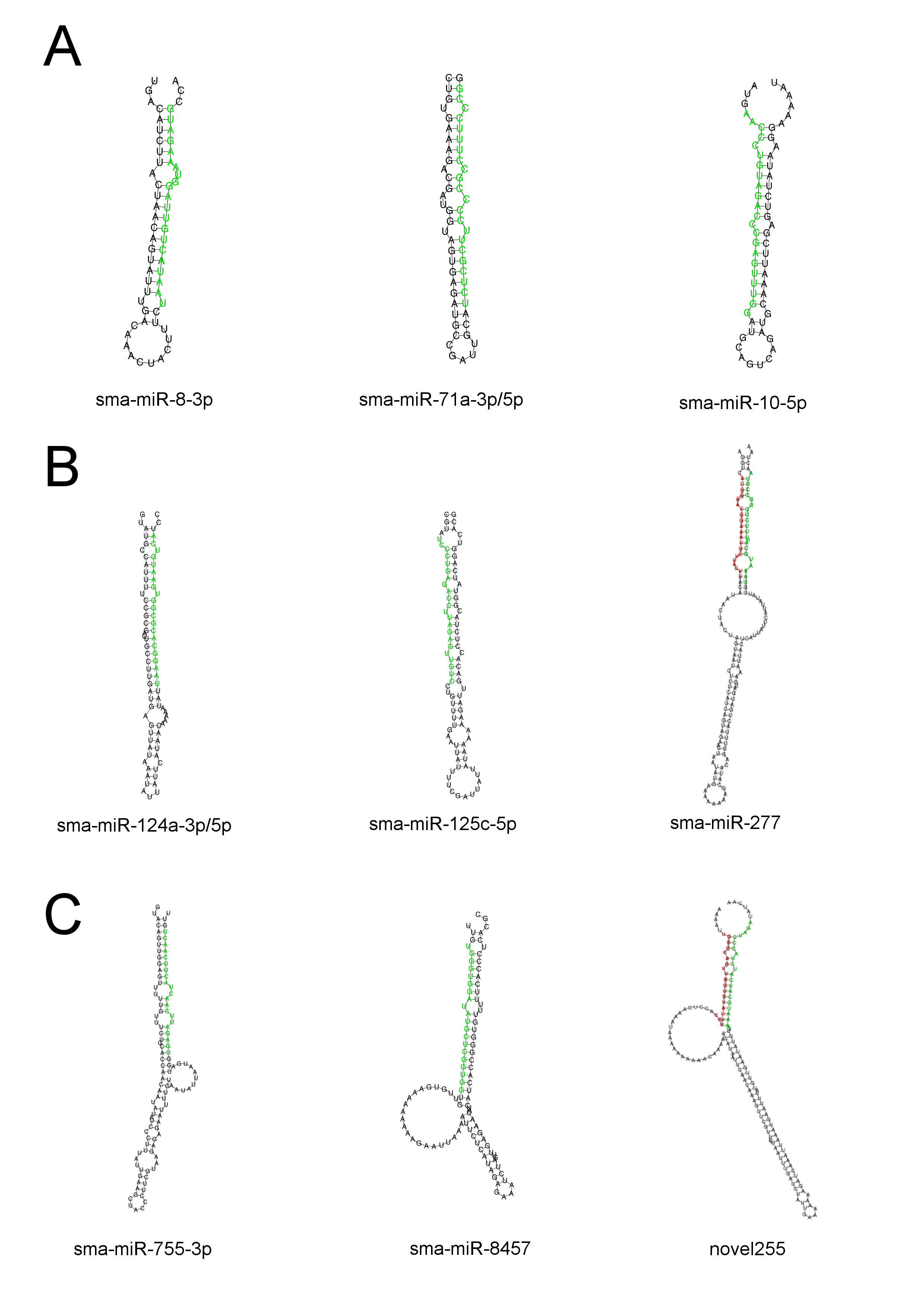
**

**Supplementary Figure 4. Stem-loop structures predicted for known and novel small RNAs in *Schistosoma mansoni.***

A) Examples of “canonical” hairpins. B and C) Examples of hairpins that required longer flanking regions to achieve a thermodynamically stable stem-loop structure. Part C depicts those with a characteristic side “bulge”.
