# Supplementary material for: MiR-277/4989 regulate transcriptional landscape during juvenile to adult transition in the parasitic helminth Schistosoma mansoni
Source: PLoS Negl Trop Dis. 2017 May 23;11(5):e0005559. doi: 10.1371/journal.pntd.0005559 (PMC5459504; doi:10.1371/journal.pntd.0005559)
Supplement: S5 Fig — Fluorescence in situ hybridization (FISH) in adult male worms using a Locked Nucleic Acid (LNA) probe to detect sma-miR-124a-3p (instead of antisense mRNA probes) and prohormone convertase 2 (pc2), that is expressed in the large number of cells in the schistosome nervous system. (A) shows detection of the expression of sma-miR-124a-3p and pc2 in the schistosome cephalic ganglia and in the nerve chords. (B) zoomed-in view of (A) showing the presence of sma-miR-124a-3p in the neural projections that connect the two cephalic ganglia (indicated with arrows) while pc2 is restricted to the cell bodies in the cephalic ganglia. (DOCX) [file pntd.0005559.s005.docx]

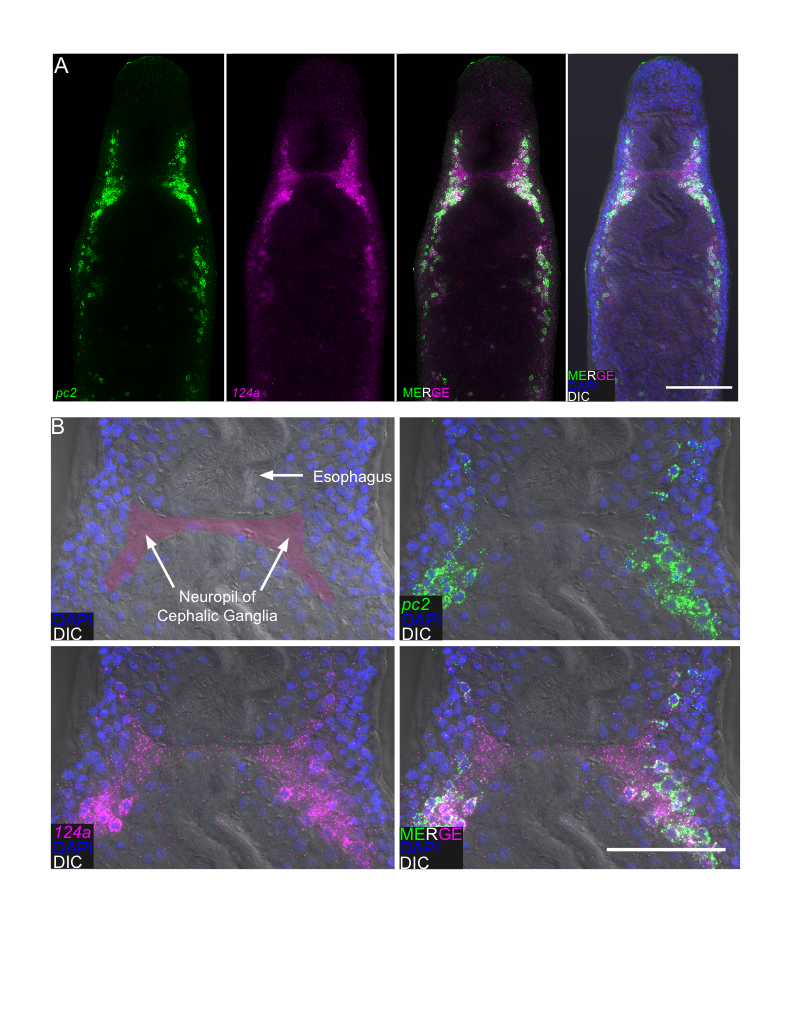


**Supplementary Figure S5. Fluorescence *in situ* hybridization of sma-miR-124.**

Fluorescence *in situ* hybridization (FISH) in adult male worms was performed as previously described [1,2] but using a Locked Nucleic Acid (LNA) probe to detect sma-miR-124a-3p (instead of antisense mRNA probes) and *prohormone convertase 2* (*pc2*), that is expressed in the large number of cells in the schistosome nervous system. (A) shows detection of the expression of sma-miR-124a-3p and *pc2* in the schistosome cephalic ganglia and in the nerve chords. (B) zoomed-in view of (A) showing the presence of sma-miR-124a-3p in the neural projections that connect the two cephalic ganglia (indicated with arrows) while *pc2* is restricted to the cell bodies in the cephalic ganglia.

1. Collins JJ, 3rd, Wang B, Lambrus BG, Tharp ME, Iyer H*, et al.* (2013) Adult somatic stem cells in the human parasite Schistosoma mansoni. Nature 494: 476-479.
2. Collins JJ, 3rd, Wendt GR, Iyer H, Newmark PA (2016) Stem cell progeny contribute to the schistosome host-parasite interface. Elife 5: e12473.
