# Supplementary material for: MiR-277/4989 regulate transcriptional landscape during juvenile to adult transition in the parasitic helminth Schistosoma mansoni
Source: PLoS Negl Trop Dis. 2017 May 23;11(5):e0005559. doi: 10.1371/journal.pntd.0005559 (PMC5459504; doi:10.1371/journal.pntd.0005559)
Supplement: S1 Text — (DOCX) [file pntd.0005559.s012.docx]

**Assay_ID Assay_Name Target**

CSS07EJ 255 sma-miR-4989(novel255)

464588_mat egr-miR-277 sma-miR-277

CSS07ET sma.U6.1.1.1 sma-U6

All Taqman rt-qPCR miRNA assays were purchased from Applied Biosystems (Life Technologies).
