# Supplementary material for: Correction: MiR-277/4989 regulate transcriptional landscape during juvenile to adult transition in the parasitic helminth Schistosoma mansoni
Source: PLoS Negl Trop Dis. 2022 Jun 2;16(6):e0007121. doi: 10.1371/journal.pntd.0007121 (PMC9162501; doi:10.1371/journal.pntd.0007121)
Supplement: S2 Text — (PDF) [file pntd.0007121.s002.pdf]

**Assay_ID**	**Assay_Name**	**Target**
CSS07EJ	255	sma-miR-4989(novel255)
464588_mat	egr-miR-277	sma-miR-277
CSS07ET	sma.U6.1.1.1	sma-U6

All Taqman rt-qPCR miRNA assays were purchased from Applied Biosystems (Life Technologies).
