# Supplementary material for: Correction: MiR-277/4989 regulate transcriptional landscape during juvenile to adult transition in the parasitic helminth Schistosoma mansoni
Source: PLoS Negl Trop Dis. 2022 Jun 6;16(6):e0010521. doi: 10.1371/journal.pntd.0010521 (PMC9170109; doi:10.1371/journal.pntd.0010521)
Supplement: S1 Fig — Odd numbers represent the test PCR while even numbers represent the “-RT” (without reverse transcriptase) control. PCR control is done against UTR of alpha tubulin Smp_090120.1. (PDF) [file pntd.0010521.s001.pdf]

Supplementary Figure S1

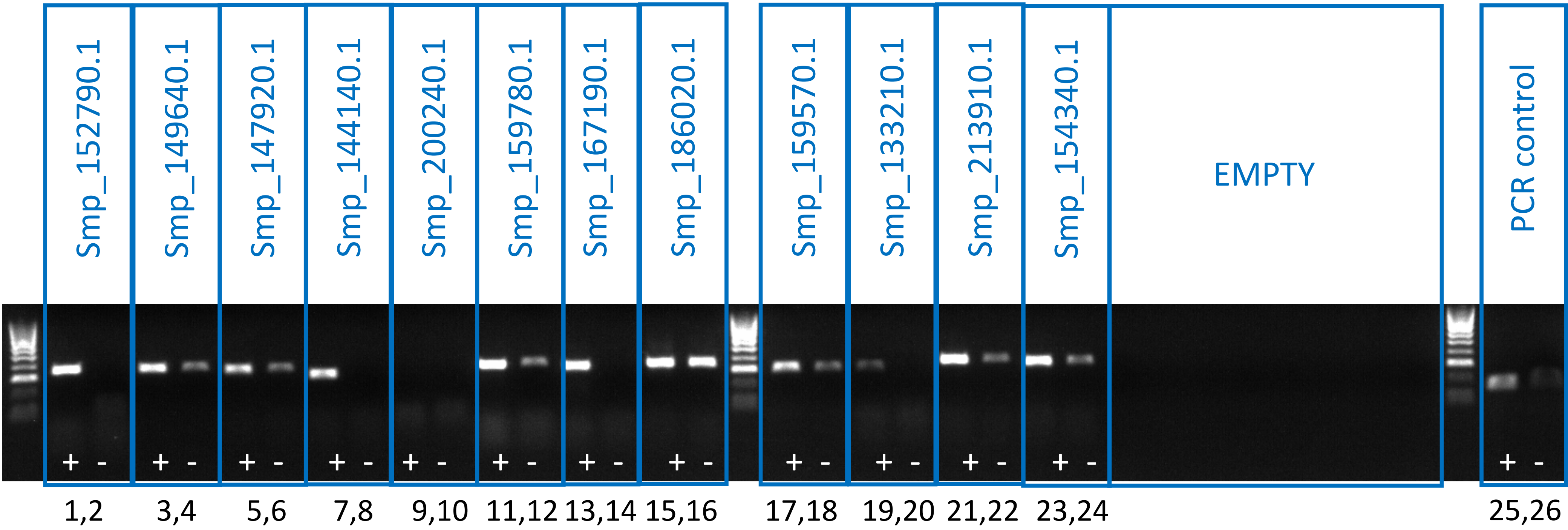

Odd numbers represent the test PCR while even numbers represent the “-RT” (without reverse transcriptase) control. PCR control is done against UTR of alpha tubulin Smp_090120.1
