# Supplementary material for: Correction: MiR-277/4989 regulate transcriptional landscape during juvenile to adult transition in the parasitic helminth Schistosoma mansoni
Source: PLoS Negl Trop Dis. 2022 Jun 6;16(6):e0010521. doi: 10.1371/journal.pntd.0010521 (PMC9170109; doi:10.1371/journal.pntd.0010521)
Supplement: S1 Text — RNA isolation using phase extraction and ETOH precipitation—suitable for samples with high carbohydrate content. (DOCX) [file pntd.0010521.s002.docx]

**Supplemetary Text 1.**

**Assay_ID Assay_Name Target**

CSS07EJ 255 sma-miR-4989(novel255)

464588_mat egr-miR-277 sma-miR-277

CSS07ET sma.U6.1.1.1 sma-U6

All Taqman rt-qPCR miRNA assays were purchased from Applied Biosystems (Life Technologies).
