# Supplementary material for: Correction: MiR-277/4989 regulate transcriptional landscape during juvenile to adult transition in the parasitic helminth Schistosoma mansoni
Source: PLoS Negl Trop Dis. 2022 Jun 6;16(6):e0010521. doi: 10.1371/journal.pntd.0010521 (PMC9170109; doi:10.1371/journal.pntd.0010521)
Supplement: S2 Text — (DOCX) [file pntd.0010521.s003.docx]

**Supplementary Text 2**

Materials and Methods section.

RNA isolation using phase extraction and ETOH precipitation.

Suitable for samples with high carbohydrate content. Modified by Dr. A.V. Protasio from Chapter 3, Kistner & Maramoros, RNA isolation using phase extraction and LiCl precipitation - Lotus japonicus Handbook - ISBN 978-1-4020-3735-1. [[1](#_ENREF_1)]

1. Heat the 2% CTAB + beta-mercaptoethanol at 65C for 2 minutes.
2. Add 500ul of the CTAB buffer to the 500ul of trizol.
3. Homogenize using a mechanical device (Turrax T10 has been tested but beads are also an option)
4. Incubate at 55C for 10 minutes with occasional mixing.
5. Add 200ul of chloroform:isoamyl alcohol (24:1)
6. Mix by shaking / use vortex
7. Incubate at RT for 3 minutes.
8. Centrifuge at 12000g for 10 minutes at 4C.
9. Transfer upper phase to a fresh tube.
10. Add 1 volume of phenol:chloroform:isoamyl alcohol (25:24:1).
11. Mix by shaking / use vortex
12. Centrifuge at 12000g for 10 minutes at 4C.
13. Transfer upper phase to a fresh tube.
14. Add 1 volume of freshly prepared 100% ETOH.
15. Incubate at -20C over-night.
16. Harvest RNA by centrifugation at max speed (~20000g) for 15 minutes at 4C.
17. Remove ETOH
18. Wash pellet with 80% ETOH
19. Centrifuge at max speed (~20000g) for 5 minutes at 4C.
20. Remove supernatant
21. Air-dry the pellet, often ~30 minutes.
22. Resuspend in DEPC-water (20-30ul)
23. Run samples in Nanodrop and Bioanalyzer.

Before staring the RNA isolation, the extraction buffer is prepared and autoclaved.

1. CTAB extraction buffer:
   1. 2% CTAB (hexadecyltrimethylammonium bromide)
   2. 100mM Tris-HCL pH 8.0
   3. 25 mM EDTA
   4. 2 M NaCl

Autoclave and let to reach at least 65C before using.

Add 20ul of beta-mercaptoethanol (Sigma # M3148) per 1ml of CTAB buffer.

Prepare 2% CTAB buffer as follows (for 100ml):

1. 2 grams of CTAB (Sigma # H6269)
2. 10ml of 1M TRis-HCl (Sanger Media)
3. 5ml of EDTA solution (Sigma #E7889)
4. 40ml of 5M NaCl (Ambion #AM9760G)
5. add DEPC-water to complete 100ml volume.
6. Optional – treat CTAB with RNAsecure (Ambion #AM7005) to completely remove RNAses. Use 40ul of RNAsecure per 1ml of 2%CTAB and heat at 65C for at least 10 minutes.
7. IMPORTANT: Preferably, samples are kept in 500ul of Trizol Solution. If samples are in RNAlater, add at least 3 volumes of PBS 1x and mix well. Centrifuge at 12000g for 3 minutes. Remove RNAlater and resuspend in 500ul of Trizol.

References:

1. Kistner, C. and M. Matamoros, *RNA isolation using phase extraction and LiCl precipitation*, in *Lotus japonicus Handbook*, A.J. Márquez and J. Stougaard, Editors. 2005, Springer Netherlands: Dordrecht. p. pp 123-124.
